# Mto2 multisite phosphorylation inactivates non-spindle microtubule nucleation complexes during mitosis

**DOI:** 10.1038/ncomms8929

**Published:** 2015-08-05

**Authors:** Weronika E. Borek, Lynda M. Groocock, Itaru Samejima, Juan Zou, Flavia de Lima Alves, Juri Rappsilber, Kenneth E. Sawin

**Affiliations:** 1 Wellcome Trust Centre for Cell Biology, Institute of Cell Biology, School of Biological Sciences, University of Edinburgh, Michael Swann Building, Max Born Crescent, Edinburgh EH9 3BF, UK; 2 Department of Bioanalytics, Institute of Biotechnology, Technische Universität Berlin, Berlin 13355, Germany

## Abstract

Microtubule nucleation is highly regulated during the eukaryotic cell cycle, but the underlying molecular mechanisms are largely unknown. During mitosis in fission yeast *Schizosaccharomyces pombe*, cytoplasmic microtubule nucleation ceases simultaneously with intranuclear mitotic spindle assembly. Cytoplasmic nucleation depends on the Mto1/2 complex, which binds and activates the γ-tubulin complex and also recruits the γ-tubulin complex to both centrosomal (spindle pole body) and non-centrosomal sites. Here we show that the Mto1/2 complex disassembles during mitosis, coincident with hyperphosphorylation of Mto2 protein. By mapping and mutating multiple Mto2 phosphorylation sites, we generate *mto2-phosphomutant* strains with enhanced Mto1/2 complex stability, interaction with the γ-tubulin complex and microtubule nucleation activity. A mutant with 24 phosphorylation sites mutated to alanine, *mto2[24A]*, retains interphase-like behaviour even in mitotic cells. This provides a molecular-level understanding of how phosphorylation ‘switches off' microtubule nucleation complexes during the cell cycle and, more broadly, illuminates mechanisms regulating non-centrosomal microtubule nucleation.

## Abstract (web summary)

In *S. pombe*, cytoplasmic microtubule nucleation, which depends on the Mto1/2 complex, ceases during mitosis. Here, Borek *et al.*, show that multisite phosphorylation of Mto1/2 during mitosis disassembles the Mto1/2 complex and prevents microtubule nucleation activity.

## Graphical abstract

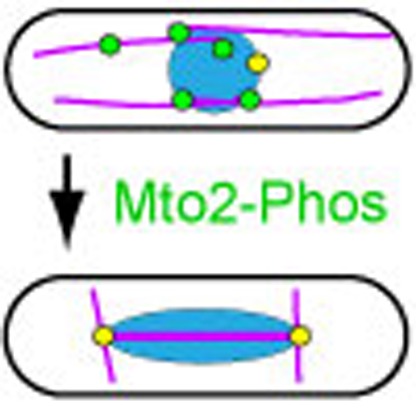

The microtubule (MT) cytoskeleton undergoes dramatic rearrangements during eukaryotic cell cycle progression and cell differentiation^1^. For example, as cells enter mitosis to form a mitotic spindle, MT nucleation from the centrosome increases several-fold^2^. The major regulator of MT nucleation *in vivo* is the γ-tubulin complex (γ-TuC; in higher eukaryotes, also called γ-tubulin ring complex, or γ-TuRC)^1^,^3^,^4^,^5^,^6^, and thus understanding the regulation of γ-TuC-dependent MT nucleation is critical to understanding MT organization from a mechanistic perspective.

In the case of the mitotic spindle, increased centrosomal MT nucleation upon mitotic onset is associated with increased centrosomal recruitment of the γ-TuC^2^,^7^,^8^. Several, possibly redundant, mechanisms have been suggested to contribute to cell cycle regulation of γ-TuC localization, and many of these involve phosphorylation of structural proteins within the centrosome by cell cycle-dependent protein kinases such as CDK1, Plk1 and Aurora A (reviewed in ref. 1). By contrast, cell cycle regulation of γ-TuC activity is less well understood, although recent advances in budding yeast indicate a role for cell cycle-dependent phosphorylation of Spc110p, a homolog of the human centrosomal protein pericentrin, in promoting mitotic spindle MT nucleation from the yeast spindle pole bodies (SPBs, yeast centrosome equivalent)^9^. Spc110p facilitates assembly of multiple γ-tubulin small complexes (γ-TuSCs)^4^ into a multimeric structure resembling the higher-eukaryotic γ-TuRC^10^,^11^,^12^, and this depends on phosphorylation of Spc110p by cell cycle kinases Cdk1p and Mps1p^9^.

Relative to the mechanisms that activate γ-TuC-dependent MT nucleation, almost nothing is known about complementary mechanisms that ‘switch off' nucleation. For example, in vertebrate cells, the Golgi apparatus is an important non-centrosomal MT organizing centre (MTOC)^13^,^14^, and during mitosis, Golgi MTOC activity is drastically decreased^15^, but the mechanistic basis for this downregulation has not yet been explored. Mechanisms that switch off MT nucleation may be of particular importance in cytoskeletal rearrangements that accompany cell differentiation—including muscle, neuronal and epithelial cell development^16^,^17^,^18^,^19^,^20^,^21^. In many of these instances, non-centrosomal MTOCs (for example, Golgi apparatus, nuclear envelope (NE), or regions of plasma membrane) can nucleate MTs alongside, or instead of, the centrosome.

Fission yeast *Schizosaccharomyces pombe* provides a particularly appropriate model system for understanding regulation of MTOCs, because it contains several distinct types of MTOCs, both centrosomal and non-centrosomal, and these vary during the cell cycle^22^ (Fig. 1a). During interphase, MTs are nucleated in the cytoplasm from the cytoplasmic face of the SPB, from the NE and from MTs themselves. Upon mitotic entry, nucleation from these interphase MTOCs ceases, and the mitotic SPBs become the only active MTOCs, nucleating intranuclear mitotic spindle MTs from the nucleoplasmic face of the SPBs. Later in mitosis, astral MTs are nucleated from the cytoplasmic face of the SPBs^23^. Finally, during cytokinesis, MTOCs are redistributed to the contractile actomyosin ring, to form a post-anaphase array of MTs^24^.

Mitotic spindle MT nucleation in fission yeast depends on the Spc110p ortholog Pcp1 (refs 25, 26), which is localized to the nucleoplasmic face of the SPB and may be regulated similarly to Spc110p^9^.

Interphase cytoplasmic MT nucleation in fission yeast, on the other hand, depends on the Mto1/2 complex, which is composed of multiple copies of the interacting proteins Mto1 and Mto2 (refs 27, 28, 29, 30, 31, 32). Multimeric Mto1/2 interacts with several copies of the γ-TuSC to generate γ-TuRC-like MT-nucleation complexes *in vivo*^32^. Mto1, like Pcp1 and Spc110p, contains a Centrosomin Motif 1 region near its N-terminus, and this is required for interaction with the γ-TuC^33^. Centrosomin Motif 1 regions are conserved in proteins involved in MT nucleation from yeast to human, and all of these appear to be specifically involved in regulation of the γ-TuC^9^,^28^,^34^,^35^,^36^.

The Mto1/2 complex itself is dynamically distributed among multiple sites during the cell cycle^24^,^27^,^28^,^29^,^30^,^31^ (see Fig. 1a for overview). During interphase, Mto1/2 localizes to SPBs and also, in the form of puncta, to interphase cytoplasmic MTs and, to a lesser extent, to the NE. During mitosis, Mto1/2 localizes to SPBs and, during later stages of division, to the contractile actomyosin ring. Mto1/2 localization to these sites is independent of interaction with the γ-TuC^33^, and thus by binding to and recruiting the γ-TuC to these sites, Mto1/2 determines the distribution of all cytoplasmic MTOC sites in the cell.

As in higher eukaryotes, relatively little is known about the molecular mechanisms that modulate MTOCs and the γ-TuC during the fission yeast cell cycle. In particular, much remains to be learned about how interphase cytoplasmic MT nucleation ceases as cells enter mitosis. Here we address this question through a combination of microscopy, biochemistry and genetics approaches. We show that Mto2 is phosphorylated on multiple sites *in vivo*, and that mitotic hyperphosphorylation of Mto2 drives disassembly of the Mto1/2 complex, leading to disruption of interaction with the γ-TuC. As part of this study, we used SILAC (stable isotope labelling of amino acids in culture) quantitative proteomics^37^,^38^ to screen for candidate mitosis-specific Mto2 phosphorylation sites, but in an indirect way, involving quantification of non-phosphorylated Mto2 peptides in mitotic versus interphase cells^39^,^40^. Overall, we find that simultaneous mutation of a large number of Mto2 phosphorylation sites to non-phosphorylatable alanine is necessary to abrogate mitotic disassembly of the Mto1/2 complex. To our knowledge, this provides the first molecular-level understanding of how interphase MT nucleation complexes are inactivated during the cell cycle and, more generally, illuminates mechanisms that regulate MT nucleation by non-centrosomal MTOCs.

## Results

### Mto1/2 complexes disassemble during mitosis

A conserved region close to the Mto1 C-terminus is both necessary and sufficient for localization to the SPB, as well as to the contractile actomyosin ring during cell division, while a nearby, less well-defined region within the Mto1 C-terminus is required for localization to MTs^24^. Recently, we described a truncated form of Mto1, ‘Mto1[NE]', in which the entire C-terminal half of Mto1 is deleted (that is, amino acids 550–1,115, which comprise all C-terminal localization regions)^32^. In *mto1[NE]* cells, puncta of the resulting Mto1/2 complex, ‘Mto1/2[NE]', become strongly enriched on the interphase NE and promote extensive MT nucleation from the NE^32^ (the underlying basis for NE localization is addressed further in the Discussion).

**Figure 1:**
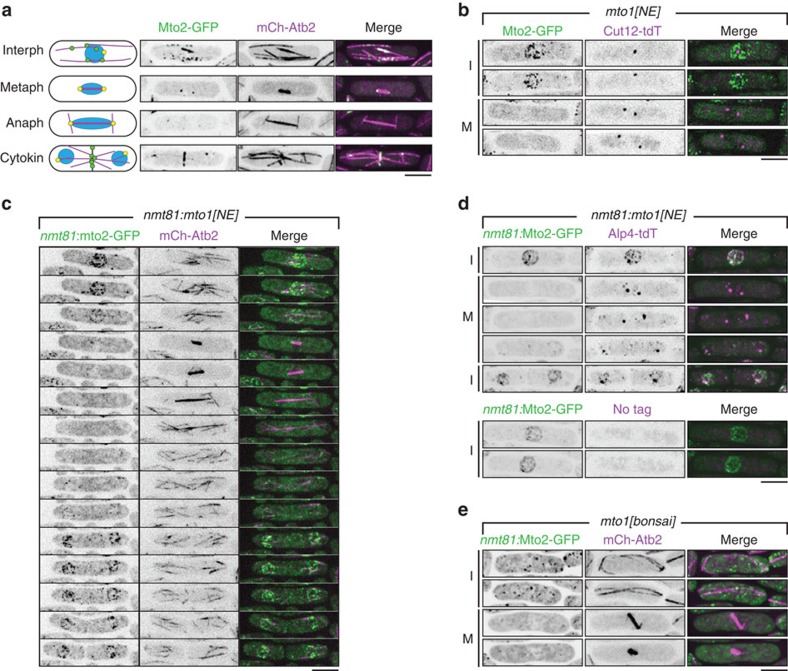
Mto1/2 complex puncta disappear during mitosis. (**a**) Cartoon summarizing dynamic distribution of Mto1/2 complex during the cell cycle (interphase, metaphase, anaphase, cytokinesis), with corresponding images of cells expressing Mto2-GFP and mCherry-tubulin (mCh-Atb2). Microtubules are shown in magenta, spindle pole bodies (SPBs) in yellow and non-SPB microtubule organizing centres containing Mto1/2 complex in green. (**b**) Localization of Mto2-GFP in *mto1[NE]* cells, together with SPB marker Cut12 fused to tandem-dimer Tomato (Cut12-tdT), in interphase (I) and mitosis (M). Note absence of Mto2-GFP puncta associated with nuclear envelope (NE) during mitosis. (**c**) Time-lapse images of cell cycle-dependent changes in localization of Mto2-GFP (mildly overexpressed from *nmt81* promoter) in *nmt81:mto1[NE]* cells, together with mCherry-tubulin (mCh-Atb2). See also Supplementary Movie 1. Sequence begins in late G2 and continues through mitosis, cytokinesis and separation of daughter cells. Interval between time points is 9 min, corresponding to every third time point of Supplementary Movie 1. (**d**) Localization of Mto2-GFP (mildly overexpressed from *nmt81* promoter) in *nmt81:mto1[NE]* cells, together with γ-TuSC protein Alp4 (homolog of mammalian GCP2 and budding yeast Spc97p) fused to tandem dimer-Tomato (Alp4-tdT) in interphase and mitosis. Lower panels show that Alp4-tdT signal at NE in interphase cells is not a result of fluorescence bleed-through. **(e)** Localization of Mto2-GFP (mildly overexpressed from *nmt81* promoter) in *mto1[bonsai]* cells, together with mCherry-tubulin (mCh-Atb2) in interphase and mitosis. Note presence of Mto2-GFP puncta in interphase and absence of puncta in mitotic cells. All images are Z-projections; individual Z-sections show NE-associated Mto1/2 complex on the NE and not in nuclear interior. Scale bars, 5 μm.

In experiments expressing Mto2-GFP in *mto1[NE]* cells, we observed a marked disappearance of Mto1/2[NE] puncta during mitosis (Fig. 1b). This was evident even in cells in which Mto1[NE] and Mto2-GFP were overexpressed (Fig. 1c,d, Supplementary Movie 1). In these cells, Mto1/2[NE] puncta were highly enriched on the NE during interphase and recruited significant amounts of the γ-TuC to the NE (imaged as γ-TuSC protein Alp4-tdTomato); however, during mitosis, these puncta also disappeared from the NE, as did the γ-TuC (Fig. 1d).

To determine whether the disappearance of Mto1/2[NE] puncta was due to cell cycle-regulated delocalization of intact Mto1/2[NE] complex puncta from the NE, or to cell cycle-regulated disassembly of the Mto1/2[NE] puncta themselves, we imaged Mto2-GFP in a different *mto1* mutant, *mto1[bonsai]*, in which Mto1 is truncated at both the N- and C-termini^32^. In *mto1[bonsai]* mutants, the resulting Mto1/2 complex, ‘Mto1/2[bonsai]', exists as free cytoplasmic puncta instead of localizing to the NE, and these puncta nucleate interphase MTs in a spatially random manner^32^. Like Mto1/2[NE] puncta, Mto1/2[bonsai] puncta disappeared during mitosis (Fig. 1e), suggesting that Mto1/2 complexes undergo cell cycle-regulated disassembly.

### Mto1/2 disassembly coincides with Mto2 hyperphosphorylation

**Figure 2:**
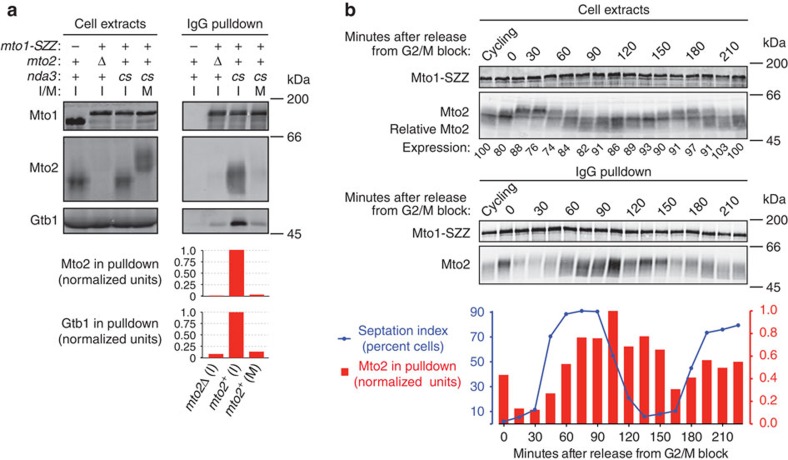
Mitotic Mto2 hyperphosphorylation coincides with disruption of Mto1–Mto2 interaction. (**a**) Anti-Mto1, anti-Mto2 and Anti-Gtb1 (*S. pombe* γ-tubulin) western blots of cell extracts and IgG pulldowns from interphase and metaphase-arrested cells expressing Mto1-SZZ (Protein A tag). Graphs show quantification of Mto2 and Gtb1 copurifying with Mto1-SZZ in the pulldowns above, normalized to the values for *mto2*^*+*^ interphase cells (central column; see Methods). I, interphase; M, mitosis; *cs*, cold-sensitive *nda3-KM311* β-tubulin allele, used for metaphase arrest. (**b**) Anti-Mto1 and anti-Mto2 western blots of cell extracts and corresponding IgG pulldowns from *mto1-SZZ cdc25-22* cells undergoing two synchronous mitoses after release from G2/M arrest. Mto2 expression levels are relative to interphase cycling cells (first lane, set to 100; see Methods). Graph shows septation index (blue line) and quantification of Mto2 copurifying with Mto1-SZZ at each time point (red bars), normalized to the 105 min time point (see Methods). Note that second mitosis is not as synchronous as the first.

To investigate this, we arrested cells in mitosis and performed pulldowns of protein A-tagged Mto1 (Mto1-SZZ) with IgG-beads. Previous western blot analysis had indicated that Mto2 migrates as multiple isoforms on SDS–polyacrylamide gel electrophoresis (SDS–PAGE), suggestive of phosphorylation^29^,^31^,^32^,^33^. We found that Mto2 isoforms from extracts of mitotically arrested cells migrated significantly more slowly than isoforms from interphase cells (Fig. 2a), suggesting that Mto2 is hyperphosphorylated during mitosis, and we subsequently confirmed this using phosphatase treatment and Phos-tag SDS–PAGE^41^ (see below). Interestingly, the amount of Mto2 copurifying with Mto1-SZZ was decreased by more than 95% in extracts from mitotic versus interphase cells (Fig. 2a). (In these experiments, the very small amount of Mto2 that did copurify with Mto1-SZZ from mitotic cells was limited to faster-migrating isoforms, which may have been generated by a small amount of dephosphorylation during sample processing or, alternatively, were present at low levels in the original cell extract.) The amount of γ-tubulin copurifying with Mto1-SZZ in extracts from mitotic cells versus interphase cells was similarly decreased, consistent with the absence of an intact Mto1/2 complex^33^.

To confirm that Mto2 hyperphosphorylation and disruption of the Mto1–Mto2 interaction were not artefacts of an extended mitotic arrest, we repeated Mto1-SZZ pulldowns in cells undergoing synchronized mitosis after release from a G2/M arrest. In these experiments, we observed hyperphosphorylation of Mto2 as cells entered mitosis and hypophosphorylation in early stages of the subsequent cell cycle (Fig. 2b), and Mto1-SZZ pulldowns revealed a negative relationship between levels of Mto2 phosphorylation and the strength of the Mto1–Mto2 interaction. In mitosis, when Mto2 was hyperphosphorylated, the Mto1–Mto2 interaction was weakest; conversely, at early stages of the next cell cycle, when Mto2 was hypophosphorylated, the Mto1–Mto2 interaction was strongest.

Collectively, these experiments establish a strong correlation between mitotic phosphorylation of Mto2 and disruption of the Mto1–Mto2 interaction, suggesting that Mto2 phosphorylation may play an important causal role in the mitotic disassembly of Mto1/2 puncta observed *in vivo*. We reasoned that the best test of this hypothesis would be to mutate phosphorylated amino-acid residues within Mto2 to non-phosphorylatable residues: if Mto2 phosphorylation were indeed a major driver of mitotic Mto1/2 complex disassembly, then Mto1/2 complex containing non-phosphorylatable Mto2 might retain interphase-like properties, such as punctum formation and, possibly, MT nucleation, in mitotic cells.

### Identifying phosphorylation sites within Mto2

**Figure 3:**
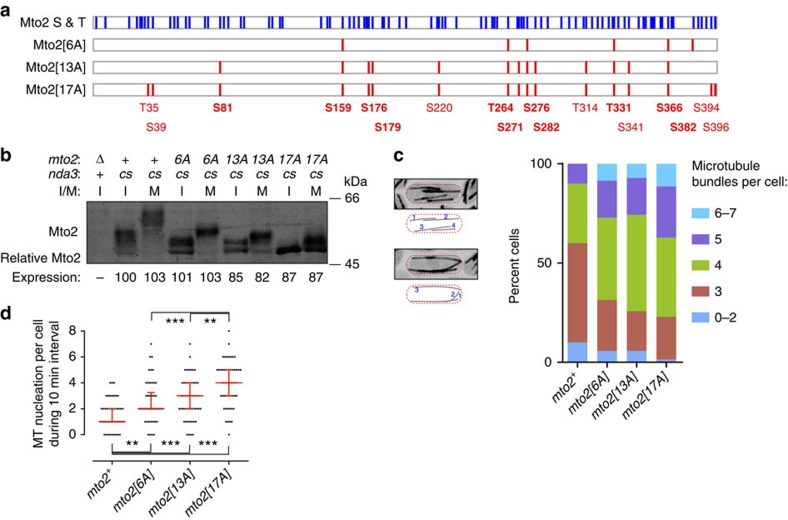
Phosphomutant Mto2 proteins are potent interphase microtubule nucleators *in vivo*. (**a**) Schematic representation showing distribution of serine and threonine residues in wild-type Mto2 (blue, total 92 residues; 14 tyrosines residues are not shown), together with sites mutated in phosphomutant Mto2 proteins (red). Mutated sites that were experimentally identified as phosphorylated are indicated in bold (these are a subset of total identified phosphosites; see Supplementary Table 1). Mutated residues are also shown in Supplementary Fig. 2. (**b**) Anti-Mto2 western blot showing altered Mto2 mobility on SDS–PAGE in *mto2-phosphomutant* cells. Mto2 expression levels are relative to interphase *mto2*^*+*^ cells (second lane, set to 100; see Methods). I, interphase; M, mitosis; *cs*, cold-sensitive *nda3-KM311* β-tubulin allele, used for metaphase arrest. (**c**) Quantification of microtubule bundle number per cell in the strains indicated, expressing GFP-tubulin. Images and cartoons at left indicate method of quantification. 70 cells were scored for each strain. *P*-values (Wilcoxon rank-sum test): *mto2*^*+*^ versus *mto2-phosphomutant* cells, *P*<1e-3. (**d**) Quantification of microtubule nucleation events per cell during a 10 min interval in the strains indicated, expressing GFP-tubulin. Fifty cells were scored for each strain. Median with interquartile range is shown in red. **P<1e-3, ***P<1e-4 (Wilcoxon rank-sum test). See also Supplementary Movie 2.

**Figure 4:**
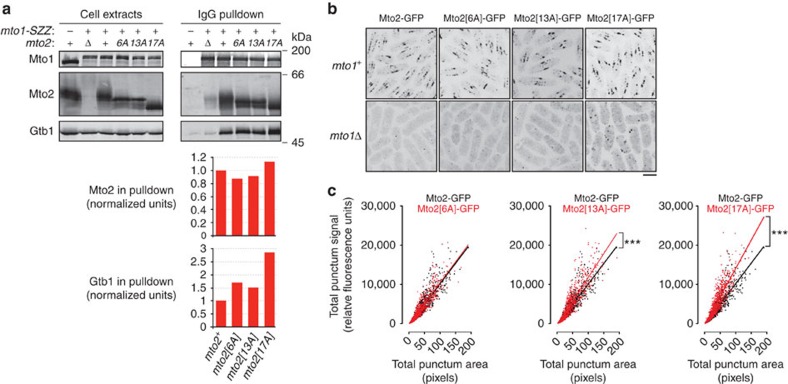
Mto1/2 complex containing phosphomutant Mto2 for ms more robust puncta *in vivo*. (**a**) Anti-Mto1, anti-Mto2 and Anti-Gtb1 Western blots of interphase cell extracts and corresponding IgG pulldowns from cells expressing Mto1-SZZ. Graphs show quantification of Mto2 and Gtb1 copurifying with Mto1-SZZ in the pulldowns above, normalized to the values for *mto2*^*+*^ cells (first column; see Methods). (**b**) Localization of wild-type and phosphomutant Mto2-GFP in *mto1*^*+*^ and *mto1Δ* cells. Z-projections. Note in particular the increased fluorescent signal of Mto2[17A]-GFP puncta in *mto1Δ* cells. (**c**) Quantification of fluorescent signals of wild-type and phosphomutant Mto2-GFP puncta, based on images similar to upper panels in (**b**). Best-fit linear regression indicates fluorescent signal per pixel of punctum area, which is related to number of Mto2-GFP molecules per (sub-resolution) volume within the puncta. ***P<1e-9 (Wilcoxon rank-sum test). Scale bar, 5 μm.

**Figure 5:**
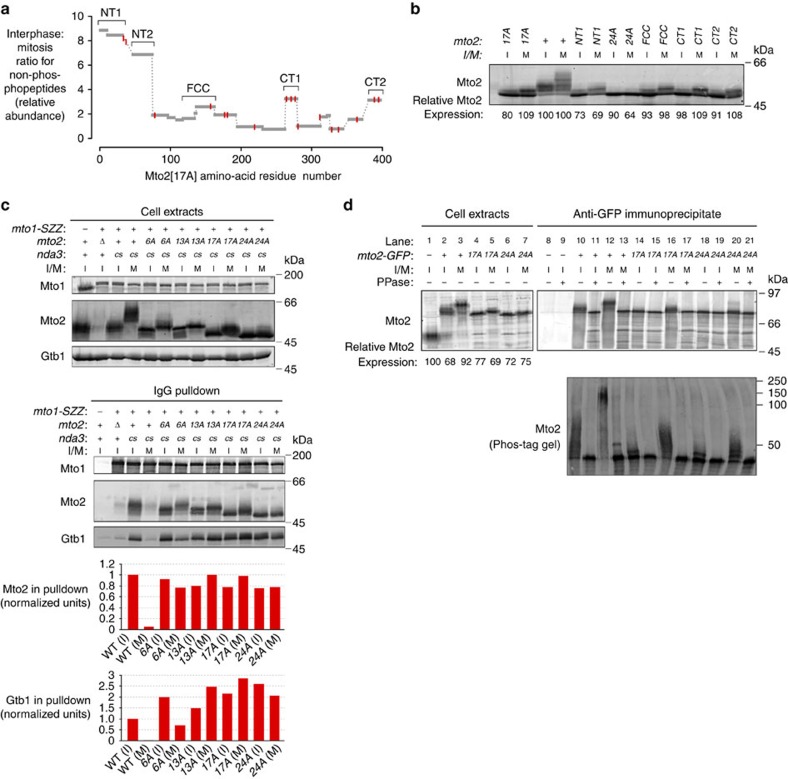
Mto2 phosphomutants can maintain Mto1–Mto2 and Mto1/2-γ-TuC interactions in mitosis. (**a**) Abundance ratios of Mto2[17A] non-phosphopeptides in interphase versus mitosis, as measured by SILAC mass spectrometry, indicating that several Mto2[17A] regions undergo high-stoichiometry modifications during mitosis. Grey bars indicate quantified peptides. Red lines indicate position of residues already mutated in Mto2[17A] (see Fig. 3a). Brackets indicate regions containing additional phosphosite mutations in the second-generation series of *mto2-phosphomutant* cells shown in (**b**). (**b**) Anti-Mto2 western blot of extracts from *mto2[17A]*, wild-type (*mto2*^*+*^) and second-generation *mto2-phosphomutant* cells, containing additional phosphosite mutations beyond those in *mto2[17A]*. Second-generation phosphomutants are named according to the region containing additional mutations, with the exception of the mutant corresponding to NT2, which is named *mto2[24A]*. Mto2 expression levels are relative to interphase *mto2*^*+*^ cells (third lane, set to 100; see Methods). Cold-sensitive *nda3-KM311* β-tubulin allele was used to arrest cells in metaphase. I, interphase; M, mitosis. (**c**) Anti-Mto1, anti-Mto2 and Anti-Gtb1 western blots of cell extracts and corresponding IgG pulldowns, from interphase and metaphase-arrested wild-type and *mto2-phosphomutant* cells expressing Mto1-SZZ. Graphs show quantification of Mto2 (or phosphomutant Mto2) and Gtb1 copurifying with Mto1-SZZ in the pulldowns above, normalized to wild-type (WT) interphase cells (first column; see Methods). *cs*, cold-sensitive *nda3-KM311* β-tubulin allele, used for metaphase arrest. (**d**) Anti-Mto2 Western blots of cell extracts and anti-GFP immunoprecipitates from untagged (*mto2*^*+*^), *mto2-GFP*, *mto2[17A]-GFP* and *mto2[24A]-GFP* cells; immunoprecipitates were either treated with lambda phosphatase (PPase, +) or untreated (−). Upper panels show proteins resolved on 10% Laemmli SDS–PAGE. Mto2-GFP expression levels are relative to cells expressing untagged Mto2 (Lane 1, set to 100; see Methods). Lower panel shows proteins resolved on 6% Phos-tag SDS–PAGE; because protein migration on Phos-tag gels is strongly affected by phosphorylation state, molecular weight markers here are only fiducial. Aspect ratio in lower panel was altered relative to original image, to match width of upper panel. All *mto2-GFP* strains are also *nda3-KM311*.

To identify phosphorylation sites within Mto2, we combined sequence analysis with experimental identification by mass spectrometry. Consistent with the prediction that Mto2 contains several intrinsically unstructured regions (Supplementary Fig. 1a), the Mto2 sequence is particularly rich in phosphorylatable residues, particularly serine (72, 20 and 14 serine, threonine and tyrosine residues, respectively, within a total of 397 amino-acid residues). Further analysis revealed at least 45 probable phosphorylation sites (NetPhos neural network algorithm)^42^ but relatively few strong consensus sites for mitotic protein kinases (for example, three sites for cyclin-dependent kinase Cdc2, three sites for Aurora kinase Ark1, and four sites for Polo kinase Plo1 (see Supplementary Table 1). We identified (uncharacterized) Mto2 homologs in *Schizosaccharomyces* species as well as in filamentous fungi, including several plant, animal and human pathogens (Supplementary Figs 1,2). However, apart from two predicted alpha-helical regions, sequence conservation across all species was very low. We therefore used a tandem 6xHis and biotinylation tag (‘HTB tag')^43^ to partially purify Mto2 from fission yeast extracts. By mass spectrometry we identified ∼20 high-confidence phosphorylation sites within Mto2 (Supplementary Fig. 3, Supplementary Table 1, Supplementary Data 1). We then combined experimental data with sequence analysis to design Mto2 mutants in which phosphorylatable residues were replaced by alanine, taking several factors into account: first, some candidate phosphorylation sites might be refractory to identification, especially if present within multi-phosphorylated peptides, which can be difficult to detect experimentally^44^,^45^. Second, not all experimentally identified phosphorylation sites are likely to be involved in cell cycle regulation of Mto1/2, because Mto2 exists in multiple phosphoisoforms even in interphase cells (Fig. 2; see also Fig. 3b, Fig. 4a, Fig. 5b–d), and also because some identified sites may be low-stoichiometry sites. Finally, simultaneous mutation of a large number of residues could be deleterious to Mto2 function.

We constructed *mto2* mutants in which 6, 13 or 17 serine or threonine codons were replaced by alanine at the endogenous *mto2* locus; we refer to the mutants as *mto2[6A]*, *mto2[13A]* and *mto2[17A]*, respectively (Fig. 3a). Most of the mutations are at [S/T]-[P] sites (6/6, 9/13 and 13/17 in *mto2[6A*], *mto2[13A]* and *mto2[17A]*, respectively; see Supplementary Table 1), which could be substrates for cyclin-dependent kinases or mitogen-activated protein kinases^46^.

Western blotting of interphase cell extracts from the *mto2-phosphomutant* strains revealed migration shifts consistent with progressively less Mto2 phosphorylation as the number of phosphosite mutations increased (Fig. 3b). In particular, on Phos-tag SDS–PAGE, migration of interphase Mto2[17A] was nearly identical to that of wild-type Mto2 treated with lambda phosphatase (see Fig. 5d, lanes 4 and 5, 14–17). By contrast, total levels of phosphomutant Mto2 protein were comparable among all strains. We conclude that the Mto2-phosphomutant proteins have significantly decreased phosphorylation *in vivo*.

### Interphase microtubule nucleation by phosphomutant Mto2

We first characterized *mto2-phosphomutant* strains during interphase, to test for any deleterious effects on Mto2 function. Surprisingly, we found that *mto2-phosphomutant* strains were in fact more competent for MT nucleation than wild-type (*mto2^+^*) cells. This was confirmed both by quantification of interphase MT bundles and by direct observation of MT nucleation in cells expressing GFP-tubulin (Fig. 3c,d, Supplementary Movie 2). In fission yeast, interphase MTs are organized into bundles that are typically composed of 2–7 individual MTs not resolvable by light microscopy^47^. Compared with wild-type cells, *mto2-phosphomutant* strains showed significantly higher percentages of cells with 4–7 bundles, and significantly lower percentages of cells with 0–3 bundles (Fig. 3c). To investigate this further, we measured MT nucleation frequency by time-lapse fluorescence videomicroscopy (Fig. 3d, Supplementary Movie 2). Compared with wild-type cells, median MT nucleation frequency was increased 2-fold in *mto2[6A]* cells, 3-fold in *mto2[13A]* cells and 4-fold in *mto2[17A]* cells.

These increases in nucleation frequency were mirrored by similar increases in the interaction of phosphomutant Mto1/2 complexes with the γ-TuC (Fig. 4a). In IgG-bead pulldowns from *mto1-SZZ* strains, the amount of γ-tubulin copurifying with Mto1-SZZ was increased ∼1.5-fold in *mto2[6A]* cells and *mto2[13A]* cells, and nearly 3-fold in *mto2[17A]* cells, relative to wild-type cells. By contrast, the amount of Mto2 copurifying with Mto1-SZZ was similar in all strains (Fig. 4a).

Efficient interaction of Mto1/2 complex with the γ-TuC is thought to require not only a binary interaction between Mto1 and Mto2 but also a multimeric form of Mto1/2 complex^32^. We thus hypothesized that the presence of phosphomutant Mto2 in the Mto1/2 complex may result in increased stability of multimeric Mto1/2, thereby leading to more stable interaction with the γ-TuC. To investigate this, we imaged cells expressing GFP-tagged phosphomutant Mto2. Like wild-type Mto2-GFP, phosphomutant Mto2-GFP in interphase cells was present at SPBs and as puncta on cytoplasmic MTs and the NE (Fig. 4b). Interestingly, phosphomutant Mto2[13A]-GFP and Mto2[17A]-GFP puncta showed both higher total fluorescent signals and higher average pixel intensities than wild-type Mto2-GFP puncta (Fig. 4c). These results are consistent with phosphomutant Mto2 leading to more stable multimeric Mto1/2 complexes in interphase cells. We also found that in an *mto1Δ* genetic background, Mto2[17A]-GFP puncta were significantly more prominent than wild-type Mto2-GFP puncta (Fig. 4b; because of the faintness and diffusion of puncta in *mto1Δ* cells, it was not possible to accurately quantify differences in puncta among these different strains). Because wild-type Mto2-GFP puncta in *mto1Δ* cells are thought to represent homo-oligomers of Mto2 (ref. 32), this suggests that the increased stability of phosphomutant Mto1/2 puncta may be due at least in part to increased interaction of Mto2 with itself. In summary, increasing the number of non-phosphorylatable residues within Mto2 leads to more stable and more active interphase Mto1/2 complexes.

### SILAC quantification of mitotic Mto2 non-phosphopeptides

On western blots, all three phosphomutant Mto2 proteins displayed slower-migrating isoforms in extracts from mitotically arrested cells compared with interphase cells (Fig. 3b). In particular, the majority of Mto2[17A] showed a discrete migration shift that was found to be due to mitosis-specific phosphorylation (see Fig. 5d, lanes 4–5, 14–17). This suggested that in spite of the effects of phosphomutant Mto2 on interphase MT nucleation, additional mitosis-specific phosphorylation may be important in regulating the Mto1/2 complex. We therefore sought to identify the relevant mitosis-specific phosphorylation sites still present in phosphomutant Mto2[17A].

We used SILAC (stable isotope labelling by amino acids in culture) mass spectrometry^37^,^38^ to compare Mto2[17A]-GFP immunopurified from interphase cells and from mitotically arrested cells (Supplementary Fig. 4). We identified ∼17 different phosphorylation sites within Mto2[17A]-GFP, and for several of these we were able to quantify relative abundance in interphase versus mitosis (Supplementary Fig. 4b, Supplementary Table 1, Supplementary Data 2). However, as a screening tool to identify candidate residues involved in mitosis-specific phosphoregulation, we focused our analysis instead on quantification of non-phosphopeptides^39^,^40^. The motivations for this are described in detail in the Supplementary Methods; here we will mention only that for a protein such as Mto2[17A], in which a significant mole fraction undergoes a mitosis-specific migration shift (Figs 3b and 5d), and most tryptic peptides contain multiple phosphorylatable residues, this approach is likely to produce the fewest ‘false-negative' results.

We obtained quantitative data for non-phosphopeptides comprising 91% of the Mto2[17A] amino-acid sequence (361/397 residues; Supplementary Data 3). This revealed five regions of Mto2[17A] for which non-phosphopeptide abundance was significantly greater in interphase versus mitotic cells (Fig. 5a), suggesting that these regions—termed NT1, NT2, FCC, CT1 and CT2—may be subject to high-stoichiometry modification in mitosis (see Supplementary Methods). In these regions several serine and threonine residues were identified as being phosphorylated (Supplementary Fig. 4b, Supplementary Table 1, Supplementary Data 2).

Taking these five regions as candidate-regions for mitosis-specific phosphorylation, we constructed a second-generation series of mutants, derived from *mto2[17A]*, in which all serines and threonines within a given region (and, in one case, one tyrosine) were mutated to alanine, in addition to the mutations already present in *mto2[17A*] (Supplementary Table 1, Supplementary Fig. 5a). Notably, the second-generation mutant protein containing phosphosite mutations in the NT2 region, which we named Mto2[24A], displayed significantly decreased mitotic phosphorylation compared to Mto2[17A] (Fig. 5b,d). By contrast, other second-generation mutant proteins (which are named according to the region mutated) were indistinguishable from Mto2[17A] in both interphase and mitosis (Fig. 5b). We therefore investigated the *mto2[24A]* mutant in more detail.

### Mto2[24A] retains interphase-like properties in mitosis

During interphase, the properties of *mto2[24A]* mutants were nearly identical to those of *mto2[17A]* mutants, in relation to MT bundle number and nucleation frequency, physical interaction of Mto1/2 with the γ-TuC, and appearance of Mto2 puncta upon GFP-tagging (Fig. 5c, Supplementary Fig. 5b–d). This indicates that Mto2[24A] is fully functional for interphase MT nucleation *in vivo* and indeed, like Mto2[17A], is a significantly ‘better' nucleator than wild-type Mto2, most likely as a consequence of increased stability of the Mto1/2 complex.

To study the properties of phosphomutant Mto2 during mitosis, we first investigated the interaction of phosphomutant Mto2[6A], Mto2[13A], Mto2[17A] and Mto2[24A] with SZZ-tagged Mto1 and the γ-TuC in interphase versus mitotically arrested cells (Fig. 5c). Interestingly, unlike wild-type cells, in all *mto2-phosphomutant* cells the amount of Mto2 copurifying with Mto1-SZZ was approximately as high during mitosis as it was during interphase. The amount of γ-tubulin copurifying with Mto1-SZZ during mitosis also remained high in *mto2[13A]*, *mto2[17A]* and *mto2[24A]* cells, again in contrast to wild-type cells. In *mto2[6A]* cells, which contain fewer phosphosite mutations, the amount of γ-tubulin copurifying with Mto1-SZZ during mitosis was decreased relative to interphase, but not as dramatically as in wild-type cells.

**Figure 6:**
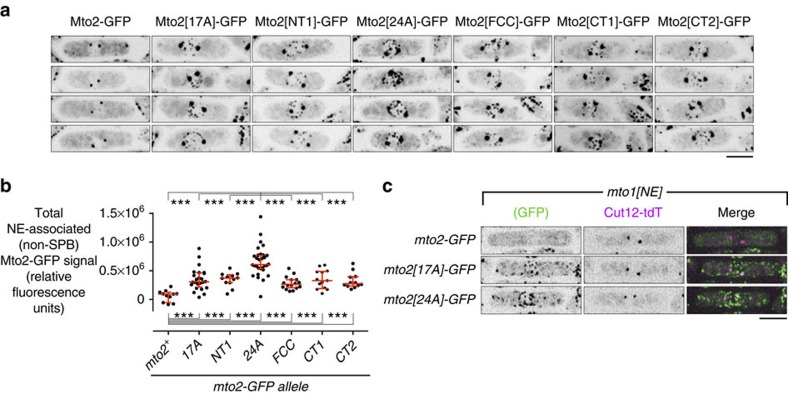
Phosphomutant Mto2[24A]-GFP forms interphase-like puncta in mitosis. (**a**) Localization of wild-type and phosphomutant Mto2-GFP, as indicated, in mitotic cells. Note robust puncta of Mto2[24A]-GFP associated with the nuclear envelope (NE) relative to other phosphomutant proteins, and absence of puncta of wild-type Mto2-GFP (except for spindle pole bodies; SPBs). (**b**) Quantification of total fluorescence signal of wild-type and phosphomutant Mto2-GFP at the NE in mitotic cells (n=11, 24, 12, 30, 16, 13 and 12 cells, respectively). Fluorescence signal at SPBs was excluded from analysis (see Methods). Median with interquartile range is shown in red. ***P<1e-4 (Wilcoxon rank-sum test). (**c**) Localization of wild-type and phosphomutant Mto2-GFP, as indicated, in mitotic *mto1[NE]* cells, together with SPB marker Cut12 fused to tandem-dimer Tomato (Cut12-tdT). Because Mto1/2 complex in *mto1[NE]* cells does not localize to SPBs, this demonstrates that NE-associated puncta of Mto2[24A]-GFP and Mto2[17A]-GFP form independently of localization to the SPB. All images are Z-projections; individual Z-sections show NE-associated Mto1/2 complex on the NE and not in nuclear interior (see Supplementary Fig. 6). Scale bars, 5 μm.

**Figure 7:**
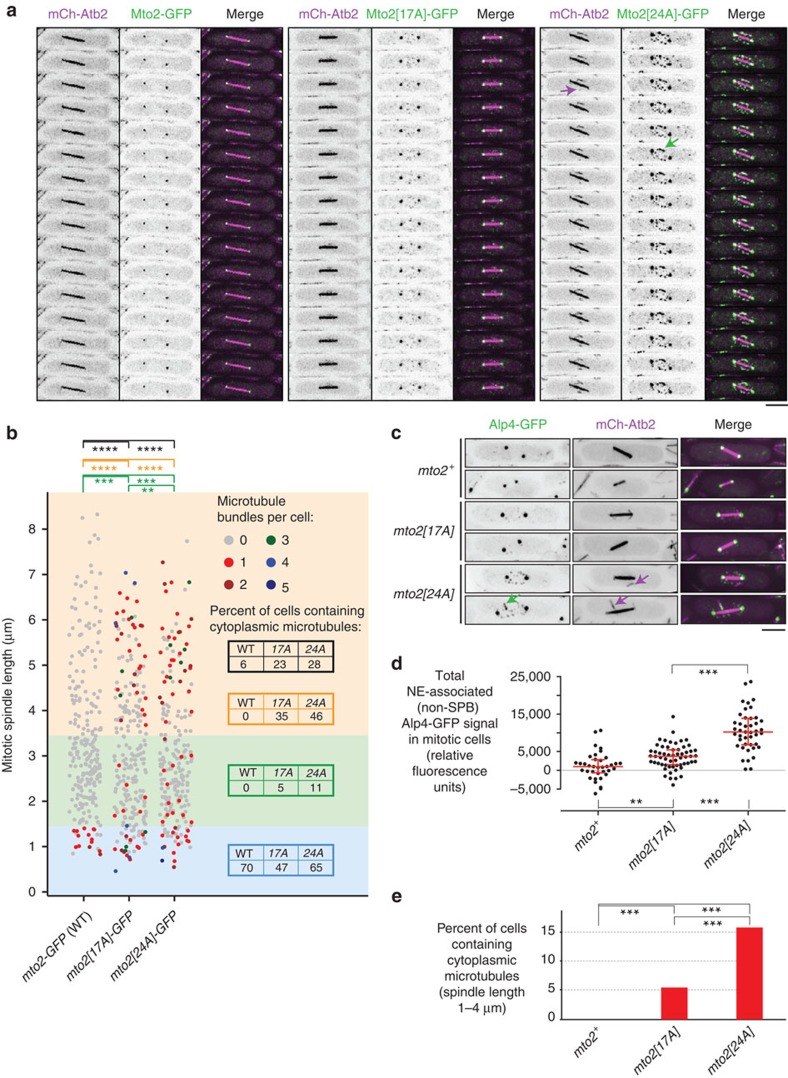
Mto2[24A] promotes interphase-like microtubule nucleation in mitosis. (**a**) Time-lapse images (1.6 s interval) of wild-type and phosphomutant Mto2-GFP in mitosis, co-imaged with mCherry-tubulin (mCh-Atb2). Magenta arrow indicates cytoplasmic microtubule (MT) nucleation event; green arrow indicates Mto2[24A]-GFP localized to MT. See Supplementary Movie 3 for corresponding movie. (**b**) Quantification of cytoplasmic MTs (mCherry-tubulin) in mitotic wild-type *mto2-GFP* (WT) and phosphomutant *mto2[17A]-GFP* and *mto2[24A]-GFP* cells (n=240, 244 and 228 cells, respectively). Data set was divided into three bins based on mitotic spindle length, roughly corresponding to prometaphase, metaphase and early-to-mid anaphase (blue, green and orange shading, respectively). Coloured tables in each bin show percent of mitotic cells within that bin containing one or more cytoplasmic MTs, for each genotype. Table in black shows this for all three bins combined. Statistical significance analyses were performed separately for each bin and are presented in corresponding colours. **P<0.05, ***P<0.01, ****P<1e-7 (Wilcoxon rank-sum test, one tailed). (**c**) Mitotic localization of γ-TuSC protein Alp4 fused to GFP (Alp4-GFP), together with mCherry-tubulin (mCh-Atb2), in wild-type and *mto2-phosphomutant* cells, as indicated. Note localization of Alp4-GFP to the nuclear envelope (NE) in *mto2[24A]* cells, and weaker localization to NE in *mto2[17A]* cells. Magenta arrows indicate cytoplasmic MTs; green arrow indicates Alp4-GFP localized to cytoplasmic MT. (**d**) Quantification of total fluorescence signal of Alp4-GFP at the NE in mitotic wild-type and *mto2-phosphomutant* cells, as indicated (*n*=36, 58 and 43 cells, respectively). Fluorescence signal at SPBs was excluded from analysis (see Methods). Median with interquartile range is shown in red. **P<1e-3, ***P<1e-9 (Wilcoxon rank-sum test, two-tailed). (**e**) Quantification of cytoplasmic MTs in mitosis in wild-type and *mto2-phosphomutant* cells expressing Alp4-GFP and mCherry-tubulin. Percent of cells containing one or more cytoplasmic MTs is shown, for cells with spindle lengths between 1 and 4 μm (*n*=126, 138 and 118 cells, respectively). ***P<0.01 (Fisher's exact test, two-tailed). All images are Z-projections; individual Z-sections show NE-associated Mto1/2 complex on the NE and not in nuclear interior. Scale bars, 5 μm.

Based on these results, we examined the localization of GFP-tagged Mto2-phosphomutant proteins during mitosis (Figs 6 and 7a, Supplementary Fig. 6). Consistent with previous work, in mitotic cells wild-type Mto2-GFP was observed only at SPBs, and no other puncta were observed^29^,^30^,^31^; Mto2[6A]-GFP showed a similar localization. In mitotic cells Mto2[13A]-GFP and Mto2[17A]-GFP were observed not only at SPBs but also occasionally as faint puncta on the NE, and Mto2[24A]-GFP was observed both at SPBs and as much more prominent puncta on the NE (Fig. 6a, Supplementary Fig. 6a; see Discussion for why these appear specifically on the NE). Quantification revealed that the non-SPB fluorescent signal of mitotic NE-associated Mto2[24A]-GFP puncta was significantly greater than that of Mto2[17A]-GFP and the four other second-generation phosphomutant Mto2-GFPs (Fig. 6b).

In these experiments we noticed that the mitotic SPB fluorescent signal of phosphomutant Mto2-GFP was also greater than that of wild-type Mto2-GFP (see, for example, Fig. 6a); this raised the possibility that mitotic NE-associated puncta of phosphomutant Mto2-GFP might be derived from fragments or remnants of SPBs. We therefore imaged Mto2[24A]-GFP and Mto2[17A]-GFP in *mto1[NE]* cells, in which the Mto1/2 complex is unable to localize to SPBs^32^. In mitotic *mto1[NE]* cells, phosphomutant Mto2-GFPs were still observed in NE-associated puncta (Fig. 6c, Supplementary Fig. 6b), demonstrating that these puncta are generated independently of any localization to SPBs.

Given these observations, we next investigated whether *mto2-phosphomutant* cells are competent for Mto1/2-dependent cytoplasmic MT nucleation during mitosis. We imaged and quantified cytoplasmic MTs (mCherry-tubulin) in mitotic *mto2-GFP*, *mto2[17A]-GFP* and *mto2[24A]-GFP* cells (Fig. 7a,b, Supplementary Movie 3). In cells with very short spindles (Fig. 7b blue zone, roughly corresponding to prometaphase), a significant proportion of cells of all genotypes contained one or more cytoplasmic MTs, indicating that at this stage of mitosis, disassembly of interphase cytoplasmic MTs is not yet complete. In cells with short spindles (Fig. 7b green zone, roughly corresponding to metaphase), 11% of *mto2[24A]-GFP* cells and 5% of *mto2[17A]-GFP* cells contained cytoplasmic MTs, while such MTs were never observed in wild-type *mto2-GFP* cells. Moreover, in cells with medium-to-long spindles (Fig. 7b orange zone, roughly corresponding to early-to-mid-anaphase), 46% of *mto2[24A]-GFP* cells and 35% of *mto2[17A]-GFP* cells contained cytoplasmic MTs, while, again, such MTs were never observed in wild-type *mto2-GFP* cells.

To confirm that the presence of cytoplasmic MTs in mitotic *mto2[24A]-GFP* cells and *mto2[17A]-GFP* cells was not dependent on GFP-tagging, we analysed untagged wild-type (*mto2^+^*), *mto2[17A]* and *mto2[24A]* cells expressing GFP-tagged γ-TuSC protein Alp4 and mCherry-tubulin (Fig. 7c-e). In contrast to the wild-type cells, nearly all mitotic *mto2[24A]* cells displayed NE-associated Alp4-GFP puncta independent of SPBs, and these were significantly brighter than the occasional equivalent puncta observed in *mto2[17A]* cells. This suggests that Mto1/2 complex containing Mto2[24A] has a stronger association with the γ-TuC during mitosis *in vivo* than does Mto1/2 complex containing Mto2[17A]. Accordingly, in *mto2[24A]* cells with short spindles, we observed one or more cytoplasmic MTs in 16% of mitotic cells, and these were associated with NE-associated Alp4-GFP puncta. Such MTs were present in only 5% of *mto2[17A]* cells, and never in wild-type (*mto2^+^*) cells.

The number of cytoplasmic MT bundles per cell observed in mitotic *mto2[24A]* cells and, to a lesser extent, *mto2[17A]* cells is striking compared with mitotic wild-type (*mto2*^+^) cells (Fig. 7b) but nevertheless not as high as in interphase cells (see, for example, Supplementary Fig. 5a). One likely reason for this may be that during mitosis, multiple additional mechanisms operate in parallel with Mto2 phosphorylation to favour intranuclear mitotic spindle assembly versus cytoplasmic MT nucleation^48^,^49^,^50^,^51^,^52^,^53^. To investigate this, we quantified cytoplasmic MTs during mitosis in wild-type, *mto2[17A]* and *mto2[24A]* cells in a temperature-sensitive *pim1-F201S* genetic background. Pim1 is the fission yeast ortholog of human RCC1, the guanine nucleotide exchange factor for Ran GTPase, required for proper nucleocytoplasmic transport^54^,^55^,^56^. At non-permissive temperature (37°C), *pim1-F201S* mutants exhibit a partial loss-of-function and have a range of defects in mitotic spindle assembly, due to decreased intranuclear localization of several proteins, including the TACC protein Alp7 and the ch-TOG protein Alp14 (refs 51, 56). For these experiments we used increased localization of GFP-tagged Polo kinase Plo1 to the SPB as an indicator of mitotic onset, as this increase occurs independently of assembly of mitotic spindle MTs^57^,^58^. Interestingly, we found that in the *pim1-F201S* background at non-permissive temperature, 43% of all mitotic *mto2[24A]* cells and 41% of all mitotic *mto2[17A]* cells contained cytoplasmic MTs, compared with 13% of all wild-type (*mto2^+^*) cells (Supplementary Fig. 7). Further examination revealed that this was largely although not exclusively because of the fact that a significant proportion of mitotic *mto2[24A]* and *mto2[17A]* cells contained unseparated SPBs, compared with wild-type cells (27, 25 and 9%, respectively; Supplementary Fig. 7). Overall, these results suggest that mitotic cytoplasmic MT nucleation in *mto2-phosphomutant* cells is limited at least in part by additional mechanisms that normally promote spindle assembly; when these mechanisms are compromised, cytoplasmic MT nucleation by phosphomutant Mto2 can exacerbate spindle assembly defects, particularly during the earliest stages of spindle assembly.

Collectively, our results indicate that *mto2-phosphomutant* cells, in particular *mto2[24A]*, retain interphase-like properties in mitotic cells. This strongly supports the hypothesis that Mto2 phosphorylation during mitosis negatively regulates the Mto1/2 complex. However, it remained formally possible, albeit unlikely, that the phenotypes of *mto2-phosphomutant* cells are due not to decreased phosphorylation but rather to nonspecific effects resulting from simultaneous mutation of multiple amino-acid residues. We therefore constructed *mto2[24D]* and *mto2[24E]* phosphomimetic mutants, in which all residues mutated to alanine in *mto2[24A]* were instead mutated to aspartate and glutamate, respectively. According to our hypothesis, phosphomimetic Mto2 would be predicted to show ‘mitotic-like' behaviour during interphase—that is, impaired function relative to wild-type interphase Mto2 and phosphomutant Mto2. Consistent with this, we found that unlike wild-type Mto2 and other phosphomutant Mto2 proteins, only negligible amounts of interphase Mto2[24D] and Mto2[24E] copurified with Mto1-SZZ, and in these cells the γ-TuC also failed to copurify with Mto1-SZZ (Supplementary Fig. 8a). In addition, GFP-tagged Mto2[24D] and Mto2[24E] formed far fewer cytoplasmic puncta compared with Mto2-GFP during interphase, and quantification of MT bundle number revealed that *mto2[24D]-GFP* and *mto2[24E]-GFP* mutants contained significantly fewer interphase MT bundles than wild-type (*mto2-GFP*) cells and indeed more closely resembled *mto2Δ* cells (Supplementary Fig. 8b,c). Thus the phenotypes of *mto2-phosphomimetic* mutants support our interpretation of *mto2-phosphomutant* phenotypes.

## Discussion

**Figure 8:**
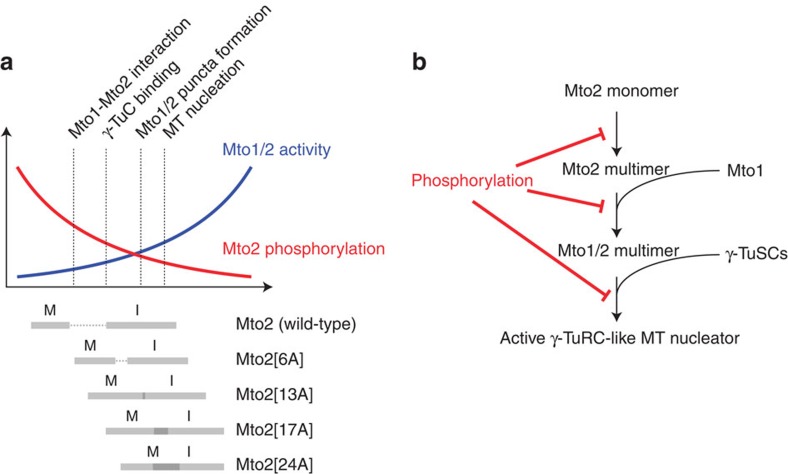
Model for regulation of Mto1/2 complex assembly and activity by phosphorylation. (**a**) Schematic diagram showing how the activity of wild-type and phosphomutant Mto1/2 complex is regulated by phosphorylation. Diagram depicts negative relationship between Mto2 phosphorylation (red) and Mto1/2-dependent microtubule (MT) nucleation (blue), aligned with range of Mto2 phosphorylation states in interphase (I) and mitosis (M) in wild-type Mto2 and Mto2 phosphomutants (grey bars). In wild-type cells, interphase levels of Mto2 phosphorylation allow efficient interaction with Mto1 and γ-TuC binding, leading to Mto1/2 complex puncta formation and microtubule (MT) nucleation; mitotic hyperphosphorylation of Mto2 abolishes these interactions, leading to suppression of microtubule nucleation. In *mto2-phosphomutant* cells, increasing the number of mutated phosphosites leads to a graded decrease in Mto2 phosphorylation and a corresponding graded increase in Mto1/2 activity in interphase and mitosis. (**b**) Role of Mto2 phosphorylation in context of model for Mto1/2 complex assembly proposed by Lynch *et al.*^32^. Several different aspects of Mto1/2 assembly and activity may be regulated by Mto2 phosphorylation in both interphase and mitosis, with higher levels of phosphorylation in mitosis. Interaction between the Mto1/2 multimer and γ-TuSCs might be regulated either directly, by altering affinity between Mto2 and γ-TuSCs, or indirectly, by altering the multimeric state of the Mto1/2 complex, thereby affecting cooperative binding to γ-TuSCs. MT, microtubule.

In spite of recent advances in understanding of mechanisms of MT nucleation *in vivo*, much remains to be learned about their regulation during the cell cycle, as well as during cell differentiation^1^,^5^,^6^. In particular, little is known about how certain modes of nucleation are ‘switched off' at the same time as other modes of nucleation are ‘switched on'. Our results here suggest a model for cell cycle regulation of cytoplasmic MT nucleation in fission yeast, in which multisite phosphorylation of Mto2 modulates the assembly and/or stability of the multimeric Mto1/2 complex (Fig. 8). During mitosis, when the intranuclear mitotic spindle is assembled, Mto2 becomes hyperphosphorylated, coincident with disassembly of the Mto1/2 complex, as demonstrated both by the disappearance of Mto2-GFP puncta *in vivo* and by the loss of physical interaction between Mto1 and Mto2, which in turn leads to loss of interaction with the γ-TuC. By mutating multiple experimentally identified and candidate phosphorylation sites within Mto2, we generated a series of phosphomutant versions of Mto2, including Mto2[24A], which has only a very low level of phosphorylation in both interphase and mitosis. During interphase, all *mto2-phosphomutant* cells nucleate more MTs than wild-type cells, and nucleation frequency increases with the number of mutations, in a graded manner. Notably, during mitosis, when Mto1/2 complexes in wild-type cells disassemble, Mto1/2 complexes in *mto2[24A]* mutant cells (and, to a lesser extent, *mto2[17A]* cells) retain interphase-like behaviour, remaining intact and capable of nucleating cytoplasmic MTs. Conversely, phosphomimetic *mto2[24D]* and *mto2[24E]* mutants show strongly impaired, ‘mitotic-like' behaviour in interphase. It is important to note that *mto2[24D]* and *mto2[24E]* phenotypes could in principle be due to nonspecific effects rather than mimicking mitotic hyperphosphorylation *per se,* particularly because it is not clear whether all 24 sites would be simultaneously phosphorylated on a single wild-type Mto2 molecule *in vivo*. However, because both Mto2[24D] and (especially) Mto2[24E] are expressed at levels similar to wild-type Mto2, and both proteins show some limited localization *in vivo*, both are likely properly folded to some extent. Furthermore, even if some *mto2-phosphomimetic* phenotypes were due in part to nonspecific effects, the fact that these phenotypes are completely different from *mto2-phosphomutant* phenotypes strongly argues against the possibility that the *mto2-phosphomutant* phenotypes themselves are due to nonspecific effects.

The *mto2[24A]* phosphomutant was generated as a result of quantifying Mto2[17A] non-phosphopeptides in interphase versus mitosis; this identified candidate-regions in which to mutate serine and threonine residues to alanine. Among the five regions mutated, the NT2 region, which is mutated in *mto2[24A]*, was the only one in which we did not directly identify phosphopeptides from Mto2[17A] by mass spectrometry (Supplementary Table 1, Supplementary Data 2). This illustrates the potential power of our approach, especially when direct mass spectrometric evidence for phosphorylation may not be forthcoming (see Supplementary Methods for further discussion). We would argue that such an approach can be valid provided it is accompanied by additional analysis (for example, western blots of Phos-tag gels). It remains formally possible that mutation within the NT2 region leads to a loss of phosphorylation elsewhere in the protein. However, given our additional finding that mutation of phosphorylatable residues in all other regions tested (NT1, FCC, CT1 and CT2) did not alter migration relative to Mto2[17A] (Fig. 5b), this could be considered unlikely.

Although both *mto2[17A]* and *mto2[24A]* mutants show strongly enhanced interphase MT nucleation, and both Mto2[24A] and Mto2[17A] proteins can physically interact with Mto1 and the γ-TuC during mitosis, *mto2[24A]* mutants display significantly stronger interphase-like behaviour during mitosis. The simplest interpretation of these results is that distributed, multisite phosphorylation of Mto2 (a protein with several predicted disordered regions) alters multiple aspects of Mto2 function in the context of the Mto1/2 protein complex (Fig. 8b), and that the cumulative effects of this are most strongly abrogated in Mto2[24A]. *In vivo*, actively nucleating Mto1/2 complex is present as higher-order multimers that interact with multiple γ-TuSCs, with an estimated ∼13 copies of Mto1 and of Mto2 in each nucleating complex^32^. Moreover, in addition to interacting with Mto1, Mto2 also interacts with itself^32^, and also possibly very weakly with the γ-TuC, independent of interaction with Mto1 (ref. 33). Although the detailed architecture of the Mto1/2 complex remains unknown, we would speculate that within this context, a gradation of levels of Mto2 phosphorylation would lead to a gradation of phenotypes, because of the quantitative effects on protein–protein interactions as well on protein conformation. In the future, this would best be investigated with purified wild-type and mutant proteins.

The cytoplasmic MT nucleation seen in mitotic *mto2[24A]* cells is not as robust as in interphase cells, and our observations in *pim1-F201S* cells suggest that this is due at least in part to additional mechanisms that normally promote intranuclear mitotic spindle assembly at the expense of cytoplasmic MT assembly. Many of the proteins that stabilize interphase cytoplasmic MTs in fission yeast also stabilize mitotic spindle MTs and thus are imported into the nucleus during mitosis; these include the TACC protein Alp7 and the ch-TOG protein Alp14 (refs 51, 52, 53), as well as the MT-bundling protein (PRC1 homolog) Ase1 (refs 49, 50). In addition, in the related filamentous fungus *Aspergillus nidulans*, there is evidence for net import of αβ-tubulin dimer into the nucleus specifically during mitosis^48^; a similar mechanism may also operate in fission yeast. Thus, even if Mto1/2 complexes containing Mto2[24A] can bind to and recruit γ-TuSCs (for example, Alp4-GFP) during mitosis, a scarcity of other factors may preclude an extensive cytoplasmic MT polymerization. This underscores the notion that disassembly of the Mto1/2 complex by Mto2 phosphorylation is likely to be one of several redundant mechanisms working in concert to switch off cytoplasmic MT polymerization during mitosis, simultaneously favouring intranuclear spindle MT polymerization.

The level of mitotic cytoplasmic MT nucleation in *mto2[24A]* mutants also provides an explanation for why mitotic puncta of Mto2[24A]-GFP (and Mto2[17A]-GFP) appear primarily on the NE, as do Alp4-GFP mitotic puncta in *mto2[24A]* cells. In wild-type interphase cells, Mto1/2 complex localizes to the NE if MTs are depolymerized by drug- or cold-treatment^28^,^29^, and in *mto1[NE]* cells, in which the MT-binding region of Mto1 is deleted, a similar localization to the NE occurs even without such treatment^32^ (Fig. 1b–d)—in other words, in the absence of MT binding, interphase Mto1/2 binds by default to the NE^32^. We propose that this also occurs for mitotic Mto1/2 containing Mto2[24A]. Although the molecular basis of Mto1/2 binding to the NE remains unknown, we consider it unlikely that mitotic Mto2[24A]-GFP puncta bind preferentially to the NE relative to MTs, because mitotic Mto2[24A]-GFP puncta were present not only on the NE but also on the few cytoplasmic MTs present in mitotic *mto2[24A]-GFP* cells, and similar results were obtained for Alp4-GFP puncta in *mto2[24A]* cells. (Fig. 7a,c, Supplementary Movie 3).

It might at first seem puzzling that *mto2[24A]* mutants do not show increased astral MT nucleation (that is, cytoplasmic MT nucleation from the SPBs) during mitosis; however, this is actually what one would predict. Although astral MT nucleation requires Mto1, it does not require Mto2 (refs 29, 30, 31), and thus astral nucleation would not be expected to be regulated by Mto2 phosphorylation.

Currently we do not know the identity of the protein kinase, or kinases, phosphorylating Mto2. Given the number and diversity of phosphosites, and the fact that Mto2 exists as multiple phosphorylated isoforms in interphase as well as mitosis, it is likely that multiple kinases are involved. It is not yet clear whether Mto2 interphase phosphorylation plays a specific modulatory role in MT nucleation, for example, at different stages of interphase, or at different subcellular sites. Preliminary observations suggest that Mto2-GFP puncta are more prominent in early stages of the cell cycle (that is, when Mto2 is hypophosphorylated), but the functional significance of this is unknown. One potential reason for multiple interphase Mto2 isoforms might be that Mto2 is differentially phosphorylated at different subcellular sites. However, we consider this unlikely, because Mto2 isoforms appear identical in wild-type, *mto1*Δ and *mto1[NE]* cells, even though Mto2 localization differs dramatically among these strains.

The presence of Mto2 homologs in filamentous fungi, including *Aspergillus*, *Neurospora* and several pathogens, suggests that disruption of interphase MT nucleation complexes by mitotic phosphorylation may be a general mechanism contributing to cell cycle-dependent regulation of MT nucleation. To date, none of these homologs has been functionally characterized. Most Mto2 phosphorylation sites lie outside highly conserved (and structured) regions, but analogous potential phosphorylation sites are easily identified in homologs (Supplementary Fig. 2). This is consistent with the notion that multisite phosphorylation of the type proposed here may result in broad region-specific effects on protein–protein interactions, rather than precise residue-specific conformational changes^59^,^60^. Given the sequence divergence among fungal Mto2 homologs, it is perhaps not surprising that Mto2 homologs have not yet been identified in higher eukaryotes. However, Mto1 homologs are present from yeast to human^6^,^9^,^28^. Thus in higher eukaryotes there may be proteins functionally equivalent to Mto2, and these could also be similarly regulated, and of potential importance particularly in relation to non-centrosomal MT nucleation.

## Methods

### Yeast strain construction and growth

Standard fission yeast methods and genetic techniques were used throughout^61^. Tagging and deletions of genes were performed using PCR-based methods^43^,^62^,^63^. Strains used in this study are listed In Supplementary Table 2.

To generate Mto2 phosphomutants expressed from the endogenous *mto2* promoter, a two-step gene replacement technique was used. For first-generation phosphomutants *mto2[6A]*, *mto2[13A]* and *mto2[17A]*, nucleotides 77–300 of the *mto2^+^* coding sequence were first replaced by the *ura4^+^* gene, using PCR-based methods, and the *ura4^+^* gene was then replaced by synthetic DNA fragments (GeneArt) encoding phosphomutant *mto2* alleles. Mutations were confirmed by DNA sequencing. For second-generation *mto2* phosphomutants *mto2[NT1], mto2[24A], mto2[FCC], mto2[CT1], mto2[CT2], mto2[24D]* and *mto2[24E],* the entire *mto2^+^* ORF was first replaced by *ura4^+^.*

For immunoprecipitation and pulldown experiments, cells were grown in YE5S rich medium. For imaging, cells were grown in EMM2 minimal medium using 5 g l^−1^ sodium glutamate instead of ammonium chloride as nitrogen source, and nutritional supplements at 175 mg l^−1^; we will refer to this as ‘minimal medium'^61^. All imaging experiments used minimal medium in which nutritional supplements, sodium glutamate and glucose were added after autoclaving.

For SILAC experiments, cells were grown in SILAC medium (EMM2 with 6 mM NH_4_Cl as nitrogen source, plus 60 mg l^−1^ L-arginine and 80 mg l^−1^ L-lysine^38^. Arginine and lysine were either unlabelled (‘light') or heavy-isotope labelled (‘heavy'). ‘Heavy' isotope-labelled amino acids were L-^13^C_6_^15^N_2_-lysine and L-^13^C_6_-arginine (Sigma Isotec). To ensure complete labelling with ‘heavy' amino acids, cells were grown in SILAC media for at least seven generations, and typically 10 generations. Cells in SILAC experiments were harvested in late log phase (OD_595_ 2.0).

For mitotic arrest, strains containing the cold-sensitive β-tubulin mutation *nda3-KM311* were grown in YE5S or SILAC medium to OD_595_ 1.0 at 30 °C, chilled in ice-water to 18 °C, and then grown at 18 °C for 6 h (YE5S) or 8 h (SILAC). OD_595_ at harvest was ∼1.8. In these experiments, asynchronous cultures grown in parallel were used as ‘interphase' cells. The small proportion of mitotic cells present in asynchronous cultures could essentially be ignored—as seen, for example, by comparing the migration of Mto2 isoforms on SDS–PAGE in interphase versus mitosis (Figs 2, 3 and 5).

For G2/M arrest-and-release, strains containing the heat-sensitive cell cycle phosphatase mutation *cdc25-22* were grown in YE5S medium in a water bath to OD_595_ 0.2 at 25 °C and then moved to the restrictive temperature (36 °C water bath). Cells were grown in 36 °C for 3 h 30 min and then released into permissive temperature (25 °C water bath). Further details are given under Biochemical Methods.

### Microscopy and image analysis

Spinning-disk confocal microscopy was performed using a Nikon TE2000 inverted microscope with a Nikon × 100/1.45 NA PlanApo objective, attached to a modified Yokogawa CSU-10 unit (Visitech) and an iXon+ Du888 EMCCD camera (Andor), controlled by Metamorph software (Molecular Devices)^64^,^65^. For simultaneous imaging of Mto2-GFP with mCherry-Atb2, and also for co-localization imaging of Mto2-GFP and Alp4-tdTomato, an Optosplit III image splitter (Cairn Research Ltd) with a T560LPXR dichroic filter (Chroma Technology) was used.

Before live-cell microscopy, cells were grown in minimal medium at 25 °C for two days, with appropriate dilution to maintain exponential growth. Imaging was performed at 25 °C in a temperature-regulated chamber built around the microscope. The only exception to this involved experiments with *pim1-F201S* temperature-sensitive mutants, in which cells were grown in YE5S medium for 2 days at 25 °C and then shifted for 3 h to 37 °C, and imaged with the temperature-regulated chamber set to 37 °C.

Cells were mounted on thin (50 μm) agarose pads containing minimal medium and 2% agarose, between a coverslip and the microscope slide and sealed with VALAP^66^. For long movies (Fig. 1c, Supplementary Movie 1) cells were mounted on a thick (∼1 mm) agarose pad, covered with a coverslip and sealed with VALAP such that a 150 μl droplet of minimal medium surrounded the pad to prevent overdrying.

Image processing was performed using Metamorph (Molecular Devices), ImageJ, Image Pro, Image-Pro Premier 3D and Photoshop (Adobe). Images were adjusted using strictly linear contrast enhancement and are presented as maximum projections of eight Z-sections spaced by 0.6 μm, containing the entire cell volume. Movies were generated using Quicktime Pro 7 software and Fireworks (Adobe). Within the same figure panel or movie, still and time-lapse images for each channel were acquired and processed identically and thus can be compared directly, except for Fig. 7a and Supplementary Movie 3, in which contrast for individual cells was adjusted identically for individual channels, but differently for each merged image, to highlight the relevant MT nucleation events.

For quantification of interphase MT bundles and MT nucleation frequency in wild-type and *mto2-phosphomutant* strains, live-cell movies of GFP-tubulin (*nmt81:*GFP-Atb2) were used. A nucleation event was defined as the appearance of a new MT that did not originate from a MT breakage event or from an existing MT bundle. Nucleation occurring on pre-existing MT bundles was excluded from the analysis because, as a result of rapid MT bundling and sliding, it cannot be quantified with complete confidence^32^.

For quantification of interphase MT bundles in cells expressing GFP-tagged wild-type, *mto2*Δ and *mto2-phosphomimetic* alleles, still images of mCherry-tubulin (mCh-Atb2) were used.

For quantification of cytoplasmic MTs in mitotic wild-type and *mto2-phosphomutant* cells, still images of mCherry-tubulin (mCh-Atb2) were used. In addition to the number of cytoplasmic MT bundles per cell, mitotic spindle length was measured, that is, the distance between duplicated SPBs. Cells in which eMTOC formation was observed or astral-like MTs were observed emanating from both SPBs were excluded from quantification. For analysis presented in Fig. 7b, the data set was divided into three bins based on spindle length, roughly corresponding to prometaphase (0–1.4 μm), metaphase (1.4–3.4 μm) and early-to-mid anaphase (3.4–8.2 μm). For analysis presented in Fig. 7e, only cells with spindle length between 1 and 4 μm were analysed.

For quantification of cytoplasmic MTs in mitotic wild-type and *mto2-phosphomutant* cells expressing Plo1-mEGFP in the *pim1-F201S* background, still images of mCherry-tubulin (mCh-Atb2) were used. Cells were grown for two days at 25 °C, then for 3 h at 37 °C. After this, cells were imaged at 37 °C during a 1 h period (that is, the total time at 37 °C did not exceed 4 h). Mitotic cells were identified by increased Plo1-mEGFP signal at the SPB, which allowed for quantification of cytoplasmic MTs in mitotic cells with ‘zero-length' spindles (that is, SPBs not yet separated). In Supplementary Fig. 7, to avoid overcrowding of data points for ‘zero-length' spindles, some data points are displayed slightly above or below the ‘zero' position on the vertical axis (not more than 0.2 μm greater or lesser). It should be noted that because of *pim1-F201S* phenotypes and differences in experimental conditions (for example, growth medium, temperature and genetic background), results from experiments with *mto2* mutants in *pim1-F201S* cells are not directly comparable with results from experiments with *mto2* mutants in *pim1^+^* cells.

For quantification of the total signal and pixel area of Mto2-GFP (or phosphomutant Mto2-GFP) puncta in interphase cells, maximum projections of entire imaging fields were generated. Integrated Morphometry Analysis (Metamorph) was used to measure the total signal and the pixel area of all puncta in the imaging field. Background signal for each imaging field (defined as average pixel intensity of three puncta-free sub-regions) was then subtracted to produce a final corrected value for the puncta signal. For each strain, three imaging fields were analysed, and the results were combined to generate plots presented in Fig. 4c.

For quantification of the total non-SPB fluorescent signal of mitotic puncta of Mto2-GFP (or phosphomutant Mto2-GFP) and Alp4-GFP at the NE (Figs 6b and 7d, respectively), maximum projections of images of mitotic cells were generated, and the NE region was selected manually and copied to a new image file. After removing the two brightest Mto2-GFP or Alp4-GFP spots (which correspond to SPBs), the integrated intensity (that is, total signal) of the NE region was measured. Background signal for the region (defined as average intensity of a puncta-free subregion, multiplied by the total number of pixels in the entire region) was then subtracted to produce a final corrected value for the total mitotic puncta signal.

Statistical analysis was performed using R (http://www.r-project.org/). Non-parametric data were analysed using Wilcoxon rank-sum test (also known as Mann–Whitney), and categorical data were analysed using Fisher's exact test.

### Biochemical methods

In all experiments except for the pulldown shown in Fig. 2b, fission yeast protein extracts were made from yeast cell powder. Cell powder was made by freezing cell pellets dropwise in liquid nitrogen, followed by cryogrinding in a Retsch RM100 mortar grinder.

Purification of Mto2-HTB before MS analysis was performed using ∼35 g of yeast cell powder. Purification was in two steps, with the first step under denaturing conditions. In the first step, powder was resuspended in denaturation buffer (6 M GuHCl, 25 mM Tris-HCl pH 7.5, 300 mM NaCl and 20 mM imidazole). Lysate was clarified by two rounds of centrifugation at 7,800*g* for 20 min at 4 °C in a Beckman Avanti J-25 centrifuge using a JA25.50 rotor. 1 ml settled bead volume of HisBind Fractogel (Novagen), previously washed with water and activated with 1 ml of 0.5 M NiSO_4_, was incubated with the clarified lysate for 3 h at 4 °C. Resin was batch-washed six times with 10 ml with wash buffer (WB; 25 mM Tris-HCl pH 7.5, 300 mM NaCl, 20 mM imidazole) containing successively lower concentrations of guanidine hydrochloride (GuHCl, two washes each with 6, 3, 1 M GuHCl), and then three times with 10 ml of WB. Protein was eluted from HisBind Fractogel in 5 ml of elution buffer (25 mM Tris-HCl pH 7.5, 300 mM NaCl, 300 mM imidazole, 1 mM DTT, 1 mM PMSF, 1 mM benzamidine, 10 μg ml^−1^ each of ‘CLAAPE' protease inhibitors (chymostatin, leupeptin, antipain, pepstatin, E64), 60 mM β-glycerophosphate, 15 mM para-nitrophenyl phosphate, 10 mM sodium orthovanadate). In the second step, ∼4.5 × 10^8^ beads of MyOne Streptavidin C1 Dynabeads (Invitrogen/Life Technologies, USA) were used. Dynabeads were washed with water and equilibrated twice with 1 ml of elution buffer. The 5 ml elution from HisBind Fractogel was added to the beads and incubated on a rotator for 2 h at 4 °C. Dynabeads were washed three times with 2 ml Tris binding buffer (50 mM Tris-HCl pH 8.0, 150 mM NaCl, 5% [v/v] glycerol, 1 mM β-mercaptoethanol, 0.2% [v/v] Triton X-100), then three times with 2 ml of TEV cleavage buffer (25 mM Tris-HCl pH 8.0, 150 mM NaCl, 0.1% [v/v] Triton X-100, 0.5 mM EDTA, 1 mm DTT). Beads were resuspended in 500 μl of TEV cleavage buffer, and 500 units of AcTEV protease (Invitrogen/Life Technologies, USA) was added. The cleavage reaction was left overnight at 4 °C. TEV cleavage product was recovered from the beads and concentrated using TCA precipitation. The protein pellet was resuspended in 30 μl 1 × Laemmli sample buffer before SDS–PAGE and Coomassie staining.

Anti-GFP immunoprecipitation of Mto2[17A]-GFP before SILAC MS analysis was performed using approximately 35 g each of ‘light' and ‘heavy' cell powder. Lysis buffer (25 mM sodium phosphate pH 7.5, 100 mM KCl, 0.5 mM EDTA, 0.2% Triton X-100, 10 μg ml^−1^ each of ‘CLAAPE' protease inhibitors, 2 mM AEBSF, 2 mM benzamidine and 2 mM PMSF), and phosphatase inhibitors (50 mM Na β-glycerophosphate, 1 mM NaF, 0.1 mM Na_3_VO_4_, 50 nM calyculin A and 50 nM okadaic acid) were added to the cell powder at a ratio of 2 ml buffer per 1 g cell powder, which produced ∼50 ml of extract (protein concentration 12 mg ml^−1^). The solution was kept on ice until fully resuspended. Cell extracts were cleared by 2 × 15 min centrifugation at 4,000*g* in a Beckman Avanti J-26 centrifuge using a JLA 8.1000 rotor. For immunoprecipitation, each 1 ml of extract was incubated with 3 × 10^7^ Protein G Dynabeads, previously covalently coupled to 1.2 μg of homemade affinity-purified sheep anti-GFP antibody using dimethyl pimelimidate. Beads were incubated with the lysate for 90 min at 4 °C, and then washed six times with the lysis buffer. Protein was eluted from beads by 15 min incubation at 50 °C in Laemmli sample buffer, resolved on 10% SDS–PAGE gel and Coomassie stained.

For Phos-tag SDS–PAGE analysis of phosphatase-treated and untreated GFP-tagged Mto2-phosphomutant protein, 5 g of cell powder was used for each strain. Cell extracts were prepared and incubated with anti-GFP beads exactly as for anti-GFP immunoprecipitations, with the exception that lysis buffer (which included phosphatase inhibitors) was added to cell powder at a ratio of 1 ml buffer per 1 g cell powder. After 30 min incubation at 4 °C, beads were washed four times with lysis buffer, resuspended in 1 mL of lysis buffer without phosphatase inhibitors, and split into two aliquots. For one aliquot, the bound protein was immediately eluted from beads by 15 min incubation at 50 °C in Laemmli sample buffer. The other aliquot was subjected to Lambda phosphatase (NEB) treatment as follows: The lysis buffer (without phosphatase inhibitors) was removed and the beads were resuspended in 50 μl of phosphatase buffer (50 mM HEPES pH 7.5, 10 mM NaCl, 2 mM DTT, 0.01% Brij 35) supplemented with 1 mM MnCl_2_, and incubated with shaking at 30 °C overnight. Beads were then washed and the protein eluted by 15 min incubation at 50 °C in Laemmli sample buffer. In Phos-tag SDS–PAGE, the following concentrations were used in the resolving gel: 375 mM Tris-HCl pH 8.8, 6% acrylamide-bisacrylamide (37.5:1 ratio), 0.1% SDS, 0.1425% APS, 0.275% TEMED, 10 μM Phos-tag-acrylamide (Wako Chemicals) and 100 mM MnCl_2_. To monitor the progress of electrophoresis, Precision Plus Protein Prestained Standards (Biorad) were used, supplemented with 7.5 mM MnCl_2_. Following electrophoresis, the gel was washed for 15 min in 100 ml of transfer buffer (10 mM CAPS pH 11.0, 10% methanol) supplemented with 1 mM EDTA, followed by a 15 min wash in transfer buffer without EDTA, and proteins were then wet-transferred to nitrocellulose membrane.

Mto1-SZZ pulldowns (except those involving G2/M arrest-and-release) were performed using 1–2 g of cell powder. Cell extracts were prepared exactly as for anti-GFP immunoprecipitations. For pulldowns, each 1 ml of extract was incubated with 5 × 10^6^ Dynabeads M270 Epoxy (Invitrogen/Life Technologies), previously covalently coupled to rabbit IgG (Sigma). Extracts were incubated with beads for 90 min at 4 °C, and then washed six times with lysis buffer. Protein was eluted from beads by 15 min incubation at 50 °C in Laemmli sample buffer, resolved on 10% SDS–PAGE gel and transferred onto a nitrocellulose membrane.

For Mto1-SZZ pulldowns after G2/M arrest-and-release, 2 l of cells were grown in a water-bath at 25 °C overnight to OD 0.2 in YE5S. Cultures were then split into seven flasks; each flask contained 250 ml of culture, a volume corresponding to two time points. One flask was kept at 25 °C (‘cycling'), while the other six were shifted to 36 °C (in water-bath) and grown for 3 h 30 min. Cultures were then returned to 25 °C by brief incubation in ice-water slurry, taking care that temperature during cooling did not fall below 25 °C, and then maintained at 25 °C in a water-bath for time-point sampling. Successive time points were taken every 15 min for 225 min. At each time point, 125 ml of culture was collected by centrifugation for 1 min 30 s at 3,500*g* at 4 °C in Megafuge 40R (Thermo Scientific) and washed with 125 ml ice-cold STOP buffer (25 mM sodium phosphate pH 7.5, 150 mM NaCl, 50 mM NaF, 10 mM EDTA, 1 mM NaN_3_). Following centrifugation (1 min 30 s at 3,500 × *g* at 4 °C, Megafuge 40 R (Thermo Scientific)) cells were resuspended in 1 ml (time points 1–7) or 1.5 ml (time points 8–15) of lysis buffer (25 mM sodium phosphate buffer pH 7.5, 100 mM KCl, 0.5 mM EDTA pH=8.0, 0.2% TritonX-100, 10 μg ml^−1^ CLAAPE, 2 mM AEBSF, 2 mM benzamidine, 2 mM PMSF, 50 mM Na β-glycerophosphate, 1 mM NaF, 0.1 mM Na_3_VO_4_, 50 nM calyculin A and 50 nM okadaic acid). The resulting cell suspension was split into two (time points 1–7) or three (time points 8–15) screw cap microfuge tubes, the cap was replaced and the tubes were centrifuged in Biofuge Pico (Heraeus) at 16,000*g* for 30 s at room temperature. The supernatant was discarded and the pellet snap frozen in liquid nitrogen. One tube of each sample was used for each pulldown. Before pulldown, 0.2 ml of lysis buffer and 1.2 ml of 0.5 mm zirconium beads (BioSpec Products) were added to frozen cell pellets, which were then disrupted by bead-beating using a Ribolyser (Hybaid), with intermittent cooling (1 × 60 s, 3 × 20 s at a speed of 4.0, with cooling on metal rack at dry ice temperature between each beating cycle). Another 0.2 ml of lysis buffer was added; the suspension was mixed and the extract was recovered and processed as above for normal Mto1-SZZ pulldowns.

For scoring septation index in these experiments, 500 μl aliquots were taken at each time point. Cells were centrifuged in a microfuge (Heraeus) at 16,000*g* for 30 s at room temperature, and the supernatant was discarded. The pellet was resuspended in ice-cold MeOH and kept at −20 °C. An amount of 5 μL of cell suspension was incubated with 5 μL Calcofluor White M2R (0.1% fluorescent brightener 28 (F3543, Sigma), 10 mM Tris-HCl pH 7.5) for 5 min at room temperature. 5 μL of cell suspension were then placed on a microscope slide and examined under a wide-field microscope using a DAPI filter set. Two hundred cells were scored and the percentage of cells containing a septum was calculated for each time point.

Western blots of cell extracts and immunoprecipitates were probed with sheep anti-Mto1 antiserum^28^, sheep anti-Mto2 antiserum^29^ and GTU-88 mouse monoclonal anti-γ-tubulin antibody (Sigma, T6557), all at 1:1,000 dilution. Anti-Mto1 and anti-Mto2 blots were further incubated with unlabelled GT-34 mouse monoclonal anti-goat antibody (Sigma, G2904), at 1:30,000 dilution, to amplify the signal, and all blots were probed with IRDye800CW donkey anti-mouse antibody (Licor, 926-32212), at 1:10,000 dilution. Blots were imaged using an Odyssey fluorescence imager (Licor) and quantified using Image Studio (Licor).

For quantification of relative Mto2 expression levels in cdc25-22 after release from G2/M arrest (Fig. 2b), the Mto2 signal in each western blot lane was divided by the Mto1 signal in the same lane, and these values were normalized to the value for interphase cycling cells (first lane, set to 100).

For quantification of expression levels of phosphomutant and phosphomimetic Mto2 relative to wild-type Mto2 (Figs 3b and 5b,d, Supplementary Fig. 8a), the Mto2 signal in each Western blot lane was divided by the signal of a nonspecific band in the same lane (as loading control), and these values were normalized to the value for wild-type interphase cells.

For quantification of wild-type, phosphomutant and phosphomimetic Mto2 and Gtb1 (γ-tubulin) in Mto1-SZZ IgG pulldowns (Figs 2a,b, 4a and 5c, Supplementary Fig. 8a), Mto2 (wild-type and mutant) and Gtb1 signals in each western blot lane were divided by the Mto1 pulldown signal in the corresponding lane, and these values were normalized to the values obtained for interphase wild-type (*mto2+)* cells, as indicated.

### Mass spectrometry analysis

For sample preparation prior to MS analysis, protein bands of Mto2 or Mto2[17A]-GFP were excised from a Coomassie-stained gel. The protein was reduced, alkylated and digested with trypsin following standard procedures^67^. Digested peptides were desalted using C18 StageTips^68^. In SILAC experiments, interphase and mitotic samples were combined after elution from C18 StageTips.

For MS analysis after Mto2-HTB purification, an LTQ-Orbitrap mass spectrometer (ThermoElectron) was coupled online to an Agilent 1100 binary nanopump and an HTC PAL autosampler (CTC Analytics). To prepare an analytical column with a self-assembled particle frit^69^, C18 material (ReproSil-Pur C18-AQ 3 μm, Dr Maisch, GmbH) was packed into a spray emitter (75-μm inner diameter, 8-μm opening, 250-mm length; New Objectives) using an air pressure pump (Proxeon Biosystems). Mobile phase A consisted of water, 5% acetonitrile, and 0.5% acetic acid. Mobile phase B consisted of acetonitrile and 0.5% acetic acid. Peptides were loaded onto the column at a flow rate of 0.7 μl min^−1^ and eluted at a flow rate of 0.3 μl min^−1^. For a 2-h gradient run, elution used a gradient from 0 to 20% B over 75 min and then from 20 to 80% B over 13 min. Fourier transform mass spectrometry spectra were recorded at 30,000 resolution, and the six most intense peaks of the MS scan were selected in the ion trap for fragmentation (normal scan; wideband activation; filling, 7.5 × 10^5^ ions for MS scan and 1.5 × 10^4^ ions for tandem mass spectrometry (MS/MS); maximum fill time, 150 ms). The dynamic exclusion time was set to 60 s.

For MS analysis after Mto2[17A]-GFP purification, peptides were analysed on Q-Exactive mass spectrometer (Thermo Fisher Scientific) coupled with a Dionex Ultimate 3,000 RSLC nano system. A column with a spray emitter (75-μm inner diameter, 8-μm opening, 250-mm length; New Objectives) was packed with C18 material (ReproSil-Pur C18-AQ 3 μm; Dr Maisch GmbH, Ammerbuch-Entringen, Germany) using an air pressure pump (Proxeon Biosystems)^69^. Mobile phase A consisted of water and 0.1% formic acid. Mobile phase B consisted of 80% ACN acetonitrile and 0.1% formic acid. Peptides were loaded onto the column with 2% B at 500 nl min^−1^ flow rate and eluted at 200 nl min^−1^ flow rate with two gradients: linear increase from 2 to 40% B in 79 min; then increase from 40 to 95% B in 11 min. The eluted peptides were directly sprayed into the mass spectrometer.

Full MS Scans were acquired on the Q-Exactive mass analyser over the range *m/z* 300–1,750 with a mass resolution of 70,000 (at *m/z* 200). The target value was 1.0E+06. The 10 most intense peaks with charge state ≥2 were fragmented in the HCD collision cell with normalized collision energy of 25%, and MS/MS spectra were with a mass resolution of 35,000 at *m/z* 200. The target value was 5.0E+05. The ion selection threshold was 2.1E+04 counts, and the maximum allowed ion accumulation times were 20 ms for full MS scans and 120 ms for MS/MS spectra. The dynamic exclusion time was set to 45 s and repeat count equal to 1.

Mass spectrometric raw files from experiments on Mto2-HTB were processed using Mascot (version 2.4.1). Searches were conducted against SwissProt database (version 2013_07) with taxonomy filter set to *Schizosaccharomyces pombe*. The search parameters were: MS accuracy, 6 p.p.m.; MS/MS tolerance, 0.6 Da; significance threshold: 0.05; fixed modifications, cysteine carbamidomethylation; variable modifications, methionine oxidation, serine, threonine and tyrosine phosphorylation; enzyme, trypsin; maximal allowed number of missed cleavages, 2. Posterior error probability of the weakest match that was accepted after manual interrogation was PEP=0.012 (Mascot score 20.82, delta score 3.4).

Mass spectrometric raw files from analyses of Mto2[17A]-GFP were processed using MaxQuant (version 1.2.2.5)^70^. Searches were conducted against an Mto2[17A]-GFP sequence generated manually. The search parameters were: MS accuracy, 20 p.p.m.; MS/MS tolerance, 0.5 Da; fixed modification, cysteine carbamidomethylation; variable modifications, methionine oxidation, *N*-acetylation of protein *N*-terminus, serine, threonine and tyrosine phosphorylation; enzyme, trypsin; maximal allowed number of missed cleavages, 2; labelling amino-acids, arginine (Arg6) and lysine (Lys8). FDR was set to 1 as the data set was considered being too small for meaningful FDR estimation using the target-decoy method. Mto2 hits were manually validated, the weakest having a posterior error probability of PEP=0.013.

XiSPEC Spectrum Viewer (http://spectrumviewer.org/) was used to visualize MS2 spectra for figure presentation.

## Additional information

**How to cite this article:** Borek, W. E. *et al.* Mto2 multisite phosphorylation inactivates non-spindle microtubule nucleation complexes during mitosis. *Nat. Commun.* 6:7929 doi: 10.1038/ncomms8929 (2015).

## Supplementary Material

Supplementary Information Supplementary Figures 1-8, Supplementary Tables 1-2, Supplementary Methods and Supplementary References

Supplementary Data 1 Mto2 phosphopeptides identified in MS analysis of Mto2-HTB purification.

Supplementary Data 2 Mto2[17A] phosphopeptides identified in MS analysis of Mto2[17A]-GFP purification.

Supplementary Data 3 Mto2 nonphosphopeptides identified in MS analysis of Mto2[17A]-GFP purification.

Supplementary Movie 1 Mto2-GFP was co-imaged with mCherry-tubulin (mCh-Atb2) throughout the cell cycle in *mto1[NE]* cells. Movie corresponds to images shown in Fig. 1c. Z-series were acquired every 3 minutes. Maximum projections of 8 Z-sections are shown. Movie plays at 6 frames per second.

Supplementary Movie 2 Wild-type and *mto2[17A]* cells expressing GFP-tubulin (*nmt81*:GFP-Atb2) were imaged. Two examples for each genotype are shown. Red arrowheads indicate a nucleation event (i.e. appearance of a new microtubule). Z-series were acquired every 5 seconds. Maximum projections of 8 Z-sections are shown. Movie plays at 6 frames per second.

Supplementary Movie 3 Wild-type and phosphomutant *mto2-GFP* cells expressing mCherry-tubulin (mCh-Atb2) were imaged. Movie corresponds to images shown in Fig. 7a. Z-series were acquired every 1.63 seconds. Maximum projections of 8 Z-sections are shown. Movie plays at 6 frames per second.
