# Supplementary material for: Mto2 multisite phosphorylation inactivates non-spindle microtubule nucleation complexes during mitosis
Source: Nat Commun. 2015 Aug 5;6:7929. doi: 10.1038/ncomms8929 (PMC4918325; doi:10.1038/ncomms8929)
Supplement: Supplementary Information — Supplementary Figures 1-8, Supplementary Tables 1-2, Supplementary Methods and Supplementary References [file ncomms8929-s1.pdf]

Supplementary Information
Borek *et al.*

a

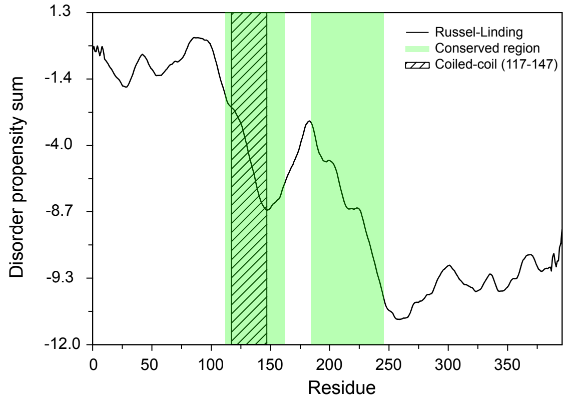

b

Organism	Accession number	Total amino acid residues	Alignment starts at residue	Alignment ends at residue
Schizosaccharomyces_pombe	NP_595204	397	1	397
Schizosaccharomyces_cryophilus	EPY50518	397	1	397
Schizosaccharomyces_octosporus	EPX71557	396	1	396
Schizosaccharomyces_japonicus	XP_002174423	463	1	437
Magnaporthe_grisea	XP_359559	1153	479	1074
Neurospora_crassa	XP_962857	1096	426	995
Gibberella_zeae	XP_387151	1170	503	1088
Penicillium_marneffei	XP_002144160	976	349	877
Coccidioides_immitis	XP_001248197	965	350	897
Paracoccidioides_brasiliensis	XP_002791015	975	317	875
Ajellomyces_capsulatus	XP_001538699	682	32	595
Penicillium_chrysogenum	XP_002562312	789	134	689
Aspergillus_nidulans	XP_662149	950	319	854
Aspergillus_clavatus	XP_001272930	1013	369	915
Aspergillus_fumigatus	XP_749412	1008	353	908
Aspergillus_terreus	XP_001218235	964	307	866
Aspergillus_flavus	XP_002379344	964	321	871

c

	S_pombe	*S_cryophilus*	*S_octosporus*	*S_japonicus*	*M_grisea*	*N_crassa*	*G_zeae*	*P_marneffei*	*C_immitis*	*P_brasiliensis*	*A_capsulatus*	*P_chrysogenum*	*A_nidulans*	*A_clavatus*	*A_fumigatus*	*A_terreus*	*A_flavus*
S_pombe	100	40.76	38.21	27.93	17.99	20.18	21.24	20.6	22.77	21.74	20.28	20.85	20.66	22.25	23.93	23.36	21.51
S_cryophilus	40.76	100	71.07	28.82	17.68	17.83	19.51	20.55	21.66	19.83	19.65	21.28	21.23	20.18	21.83	22.87	21.86
S_octosporus	38.21	71.07	100	25.88	18.65	17.14	19.88	22.15	23.15	21.35	20.87	21.35	22.22	21.45	24.26	22.35	21.92
S_japonicus	27.93	28.82	25.88	100	18.33	19.61	18.97	19.37	18.36	20.38	20.91	22.04	21.59	22.07	23.69	21.1	21.01
M_grisea	17.99	17.68	18.65	18.33	100	44.93	46.35	28.95	28.23	32.74	29.55	28.92	29.07	29.04	30.18	29.88	31.03
N_crassa	20.18	17.83	17.14	19.61	44.93	100	47.23	30.6	31.12	33.54	31.21	32.69	32.4	33.55	34.67	33.12	32.62
G_zeae	21.24	19.51	19.88	18.97	46.35	47.23	100	31.33	32.26	32.39	32.14	32.71	32.78	32.51	33.47	32.12	31.89
P_marneffei	20.6	20.55	22.15	19.37	28.95	30.6	31.33	100	43.11	43.43	41.44	44	43.56	44.82	45.21	42.19	42.26
C_immitis	22.77	21.66	23.15	18.36	28.23	31.12	32.26	43.11	100	50.38	47.96	41.67	40.24	45.22	41.95	43.7	40.78
P_brasiliensis	21.74	19.83	21.35	20.38	32.74	33.54	32.39	43.43	50.38	100	71.72	42.52	41.22	45.03	43.87	44.47	44.08
A_capsulatus	20.28	19.65	20.87	20.91	29.55	31.21	32.14	41.44	47.96	71.72	100	42.53	41.65	45.92	44.03	44.05	43.48
P_chrysogenum	20.85	21.28	21.35	22.04	28.92	32.69	32.71	44	41.67	42.52	42.53	100	52.53	51.87	52.41	53.9	52.83
A_nidulans	20.66	21.23	22.22	21.59	29.07	32.4	32.78	43.56	40.24	41.22	41.65	52.53	100	55.21	58.06	61.25	61.3
A_clavatus	22.25	20.18	21.45	22.07	29.04	33.55	32.51	44.82	45.22	45.03	45.92	51.87	55.21	100	70.33	60.66	63.06
A_fumigatus	23.93	21.83	24.26	23.69	30.18	34.67	33.47	45.21	41.95	43.87	44.03	52.41	58.06	70.33	100	62.03	62.2
A_terreus	23.36	22.87	22.35	21.1	29.88	33.12	32.12	42.19	43.7	44.47	44.05	53.9	61.25	60.66	62.03	100	66.67
A_flavus	21.51	21.86	21.92	21.01	31.03	32.62	31.89	42.26	40.78	44.08	43.48	52.83	61.3	63.06	62.2	66.67	100

Supplementary Figure 1. Identification of Mto2 homologs.
(a) GlobPlot (Supplementary Ref. 1) of Mto2 amino-acid sequence (397 residues), showing regions predicted to be disordered (upward slope) or ordered (downward slope). Regions conserved in alignment of sequence homologs (Supplementary Fig. 2) are highlighted in green. Hatched region indicates a short predicted coiled-coil.
(b) Species names and GenBank accession numbers of representative Mto2 sequence homologs identified by PSI-BLAST (Supplementary Ref. 2). Because most Mto2 homologs contain N- or C-terminal extensions (e.g. *M. grisea*, *N. crassa*, etc.), the specific amino-acid residues used to generate the alignment are also indicated.
(c) Percentage identity between individual Mto2 homologs (Clustal Omega alignment; Supplementary Ref. 3). Black indicates comparisons among *Schizosaccharomyces* species, green indicates comparisons among filamentous fungi, and red indicates comparison between *Schizosaccharomyces* species and filamentous fungi. Note that conservation between *Schizosaccharomyces* species and filamentous fungi is low (18-24%) compared to conservation within filamentous fungi (typically 30-60%).

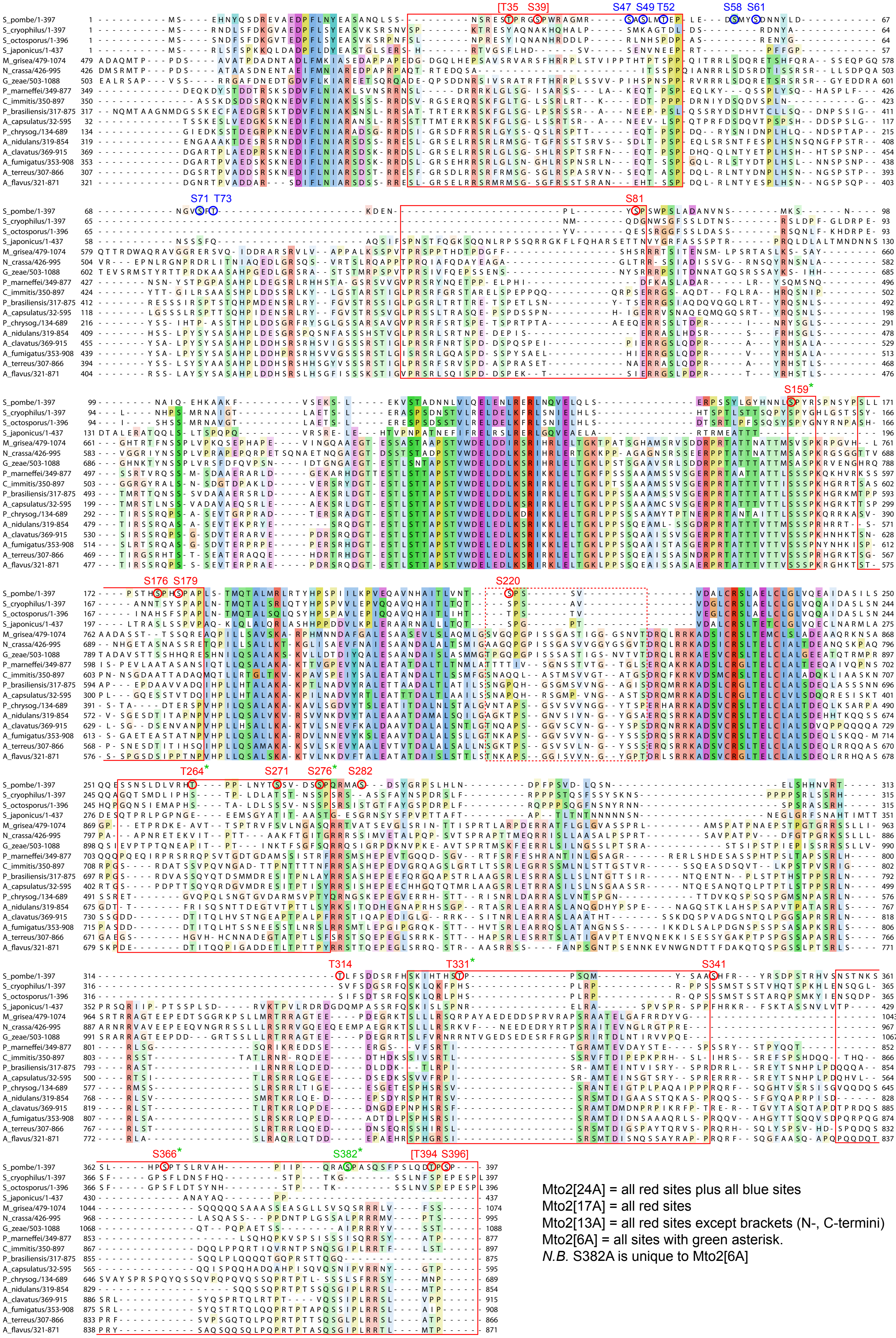

Mto2[24A] = all red sites plus all blue sites

Mto2[17A] = all red sites

Mto2[13A] = all red sites except brackets (N-, C-termini)

Mto2[6A] = all sites with green asterisk.

N.B. S382A is unique to Mto2[6A]

Supplementary Figure 2. Alignment of Mto2 homologs and position of phosphosite mutations.
Clustal Omega alignment (Supplementary Ref. 3) of fission yeast *S. pombe* Mto2 with homologs from other *Schizosaccharomyces* species and from filamentous fungi. Circled amino-acid residues indicate residues mutated to alanine in various *mto2-phosphomutant* strains; the labeling scheme used to distinguish different mutants is shown in figure. Boxed regions indicate cases in which one or more [S/T]-[P] sites present in *S. pombe* Mto2 are also present in the same general neighborhood in homologs; as is often the case for unstructured proteins (Supplementary Ref. 4), positioning of these sites is not highly conserved in the alignment. Boundaries of boxes indicate the range of candidate-similar [S/T]-[P] sites in the full collection of homologs. Box with dashed line shows an [S/T]-[P] site conserved in *Schizosaccharomyces* species but not in most other organisms.

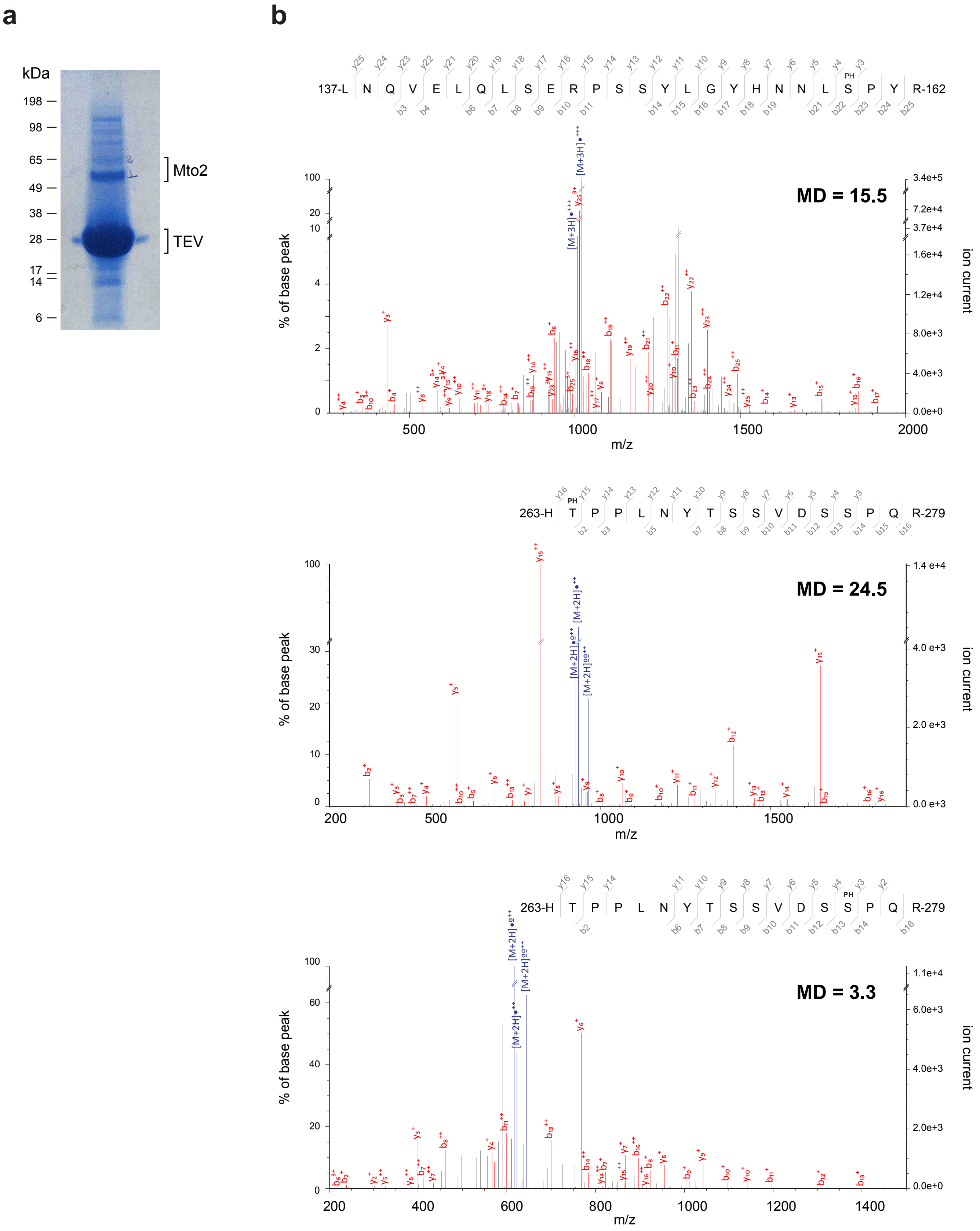

Supplementary Figure 3. Identification of Mto2 phosphorylation sites by mass spectrometry.
(a) Coomassie-blue stain of purified Mto2 (purification of Mto2-HTB) after TEV cleavage. The Mto2 band was excised prior to digestion and MS analysis.
(b) Representative spectra of Mto2 peptides containing high-confidence phosphorylation sites (see also Supplementary Data 1). Phosphorylated Ser159 was identified in the peptide LNQVELQLSERPSSYLGYHNNL**S**PYR. Phosphorylated Thr264 and Ser276 were identified separately in two different forms of the peptide H**T**PPLNYTSSVDS**S**PQR. Precursor ions are shown in blue, and fragment ions in red. **• -** indicates loss of phosphate; º - indicates loss of water. MD, Mascot Delta Score.

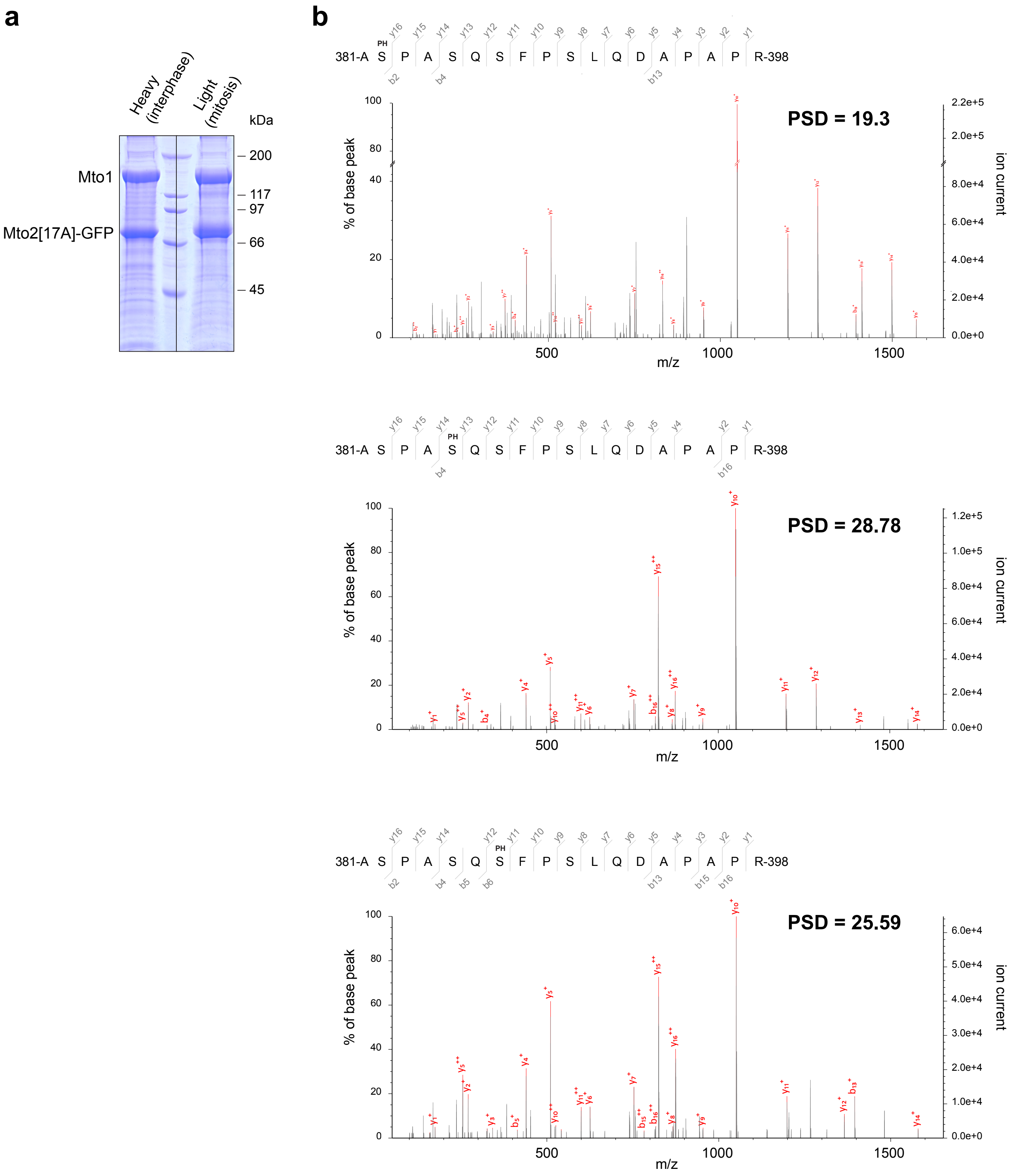

Supplementary Figure 4. Identification of Mto2[17A] phosphorylation sites by mass spectrometry.
(a) Coomassie-blue stain of purified Mto2-GFP (anti-GFP purification). Interphase and mitotic Mto2[17A]-GFP bands were excised prior to digestion and MS analysis.
(b) Example spectra of an Mto2[17A] peptide, A**S**PA**S**Q**S**FPSLQDAPAPR, in which three distinct phosphorylation sites (Ser382, Ser385 and Ser387) were identified with high confidence, each in a separate monophosphopeptide. Note that Pro397 is the last amino acid of Mto2[17A] itself; Arg398 is the first amino acid of a linker (RIPGLIN) between Mto2[17A] and GFP. See also Supplementary Data 1. PSD, Phospho (STY) Score Difference for the indicated residue (Ser382, Ser385 and Ser387, respectively).

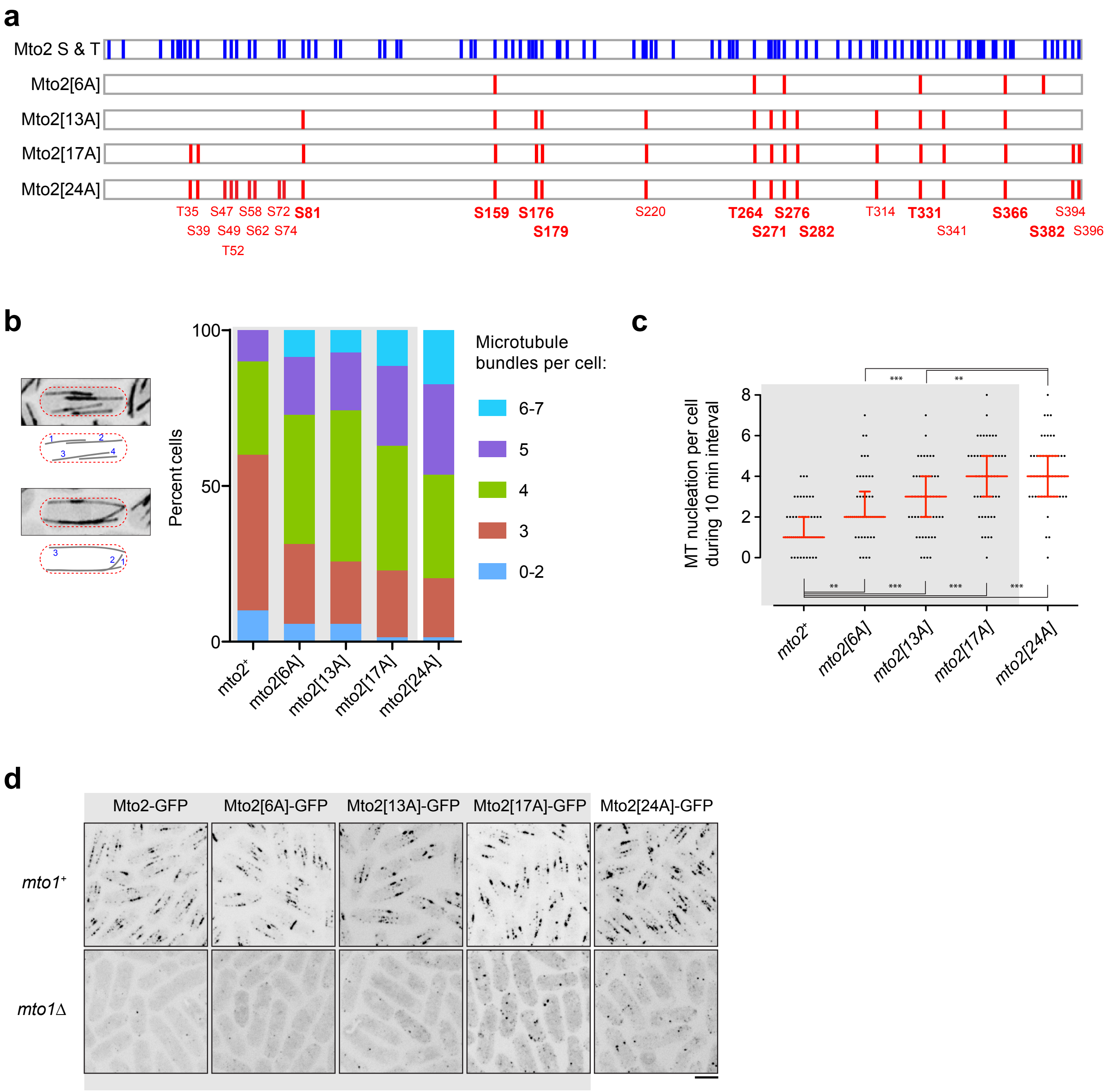

Supplementary Figure 5. Mto2[24A]-GFP and Mto2[17A]-GFP have similar functional properties during interphase.
(a) Schematic showing distribution of serine and threonine residues in wild-type Mto2 (blue, total 92 residues; 14 tyrosines residues are not shown), together with sites mutated in phosphomutant Mto2 proteins (red). Mutated sites that were experimentally identified as phosphorylated are indicated in bold (these represent a subset of total identified phosphosites; see Supplementary Table 1). Mutated residues are also shown in sequence alignment in Supplementary Fig. 2.
(b) Quantification of microtubule bundle number per cell, in the strains indicated, expressing GFP-tubulin (*nmt81*:GFP-Atb2). For each strain, 70 cells were scored. Images and cartoons at left indicate method of quantification. P values (Wilcoxon rank-sum test): *mto2+* vs *mto2-phosphomutant*, p<1e-3; *mto2[24A]* vs *mto2[13A]* and *mto2[6A]*, p<0.02. Gray box shows data reproduced from Fig. 3c, for comparison.
(c) Quantification of microtubule nucleation events per cell during a 10 min interval, in the strains indicated. For each strain, 50 cells were scored. Median with interquartile range is shown in red. ** p<1e-3, *** p<1e-4 (Wilcoxon rank-sum test). Gray box shows data reproduced from Fig. 3d, for comparison.
(d) Images showing localization of wild type and phosphomutant Mto2-GFP in *mto1*+ and *mto1*Δ cells. Z-projections. Gray box shows data reproduced from Fig. 4b, for comparison.
Scale bar, 5 μm.

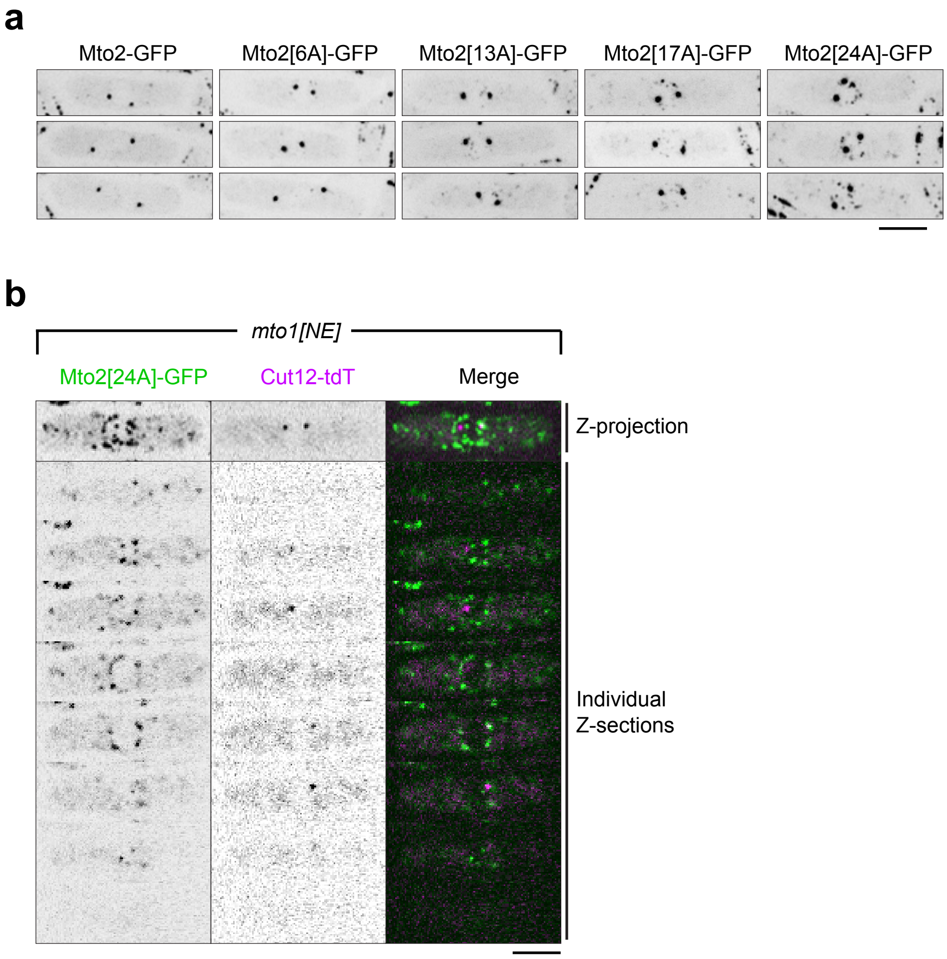

Supplementary Figure 6. Mitotic Mto2[24A]-GFP forms puncta that localize to the nuclear envelope.
(a) Localization of wild-type and phosphomutant Mto2-GFP, as indicated, in mitotic cells. Z-projections. Note absence of nuclear envelope (NE)-associated puncta of wild-type Mto2-GFP and Mto2[6A]-GFP, weak puncta of Mto2[13A]-GFP and Mto2[17A]-GFP, and more robust puncta of Mto2[24A]-GFP. All proteins localize to spindle pole bodies (SPBs).
(b) Z-projection (top row) and individual Z-sections (other rows) of the *mto1[NE]* mitotic cell shown in Fig. 6c, expressing Mto2[24A]-GFP together with SPB marker Cut12 fused to tandem-dimer Tomato (Cut12-tdT). Individual Z-sections demonstrate that Mto2[24A]-GFP localizes to the nuclear envelope and not in the nuclear interior. Contrast settings are different between the Z-projection and individual Z-sections.
Scale bars, 5 μm.

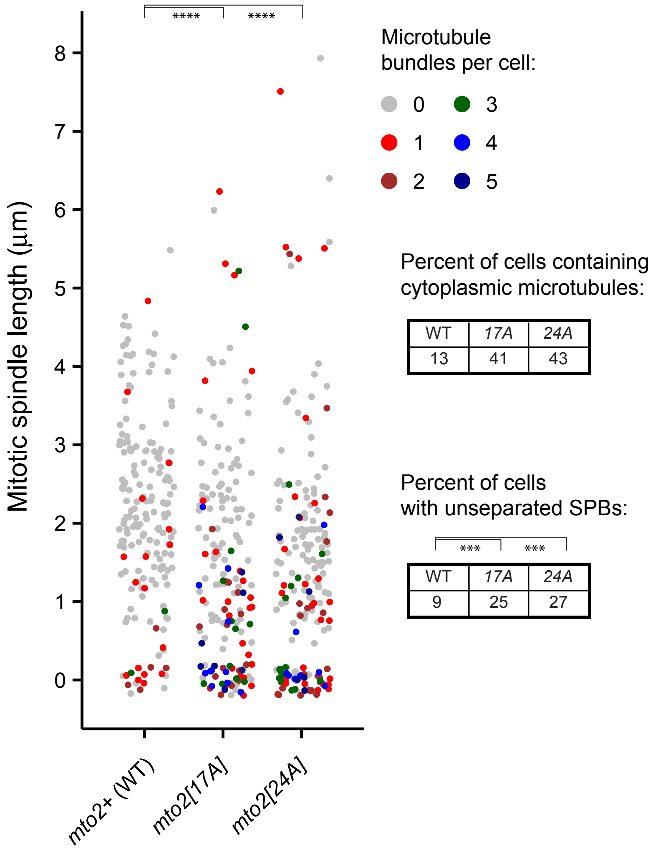

Supplementary Figure 7. Impaired nucleocytoplasmic transport due to *pim1* mutation leads to increased cytoplasmic microtubules and defects in spindle pole body separation in mitotic *mto2-phosphomutant* cells.
Quantification of cytoplasmic MTs (mCherry-tubulin) in mitotic wild-type (*mto2+*) and phosphomutant *mto2[17A]* and *mto2[24A]* cells, in *pim1-F201S* mutant genetic background at 37°C (n=182, 177 and 186 cells, respectively). Bright Plo1-GFP signal at spindle pole bodies (SPBs) was used to identify mitotic cells independent of spindle assembly state. Note that “minimum spindle length” is 0 µm (i.e. unseparated SPBs), but to avoid overcrowding of datapoints, some datapoints are displayed slightly above and below the zero position on the vertical axis. Tables show percent of mitotic cells containing one or more cytoplasmic MTs, and percent of cells in which bright Plo1-GFP signal was observed as a single spot, indicating unseparated SPBs. *** $p<1e-4$ (Fisher’s exact test), **** $p<1e-9$ (Wilcoxon rank-sum test, one-tailed).

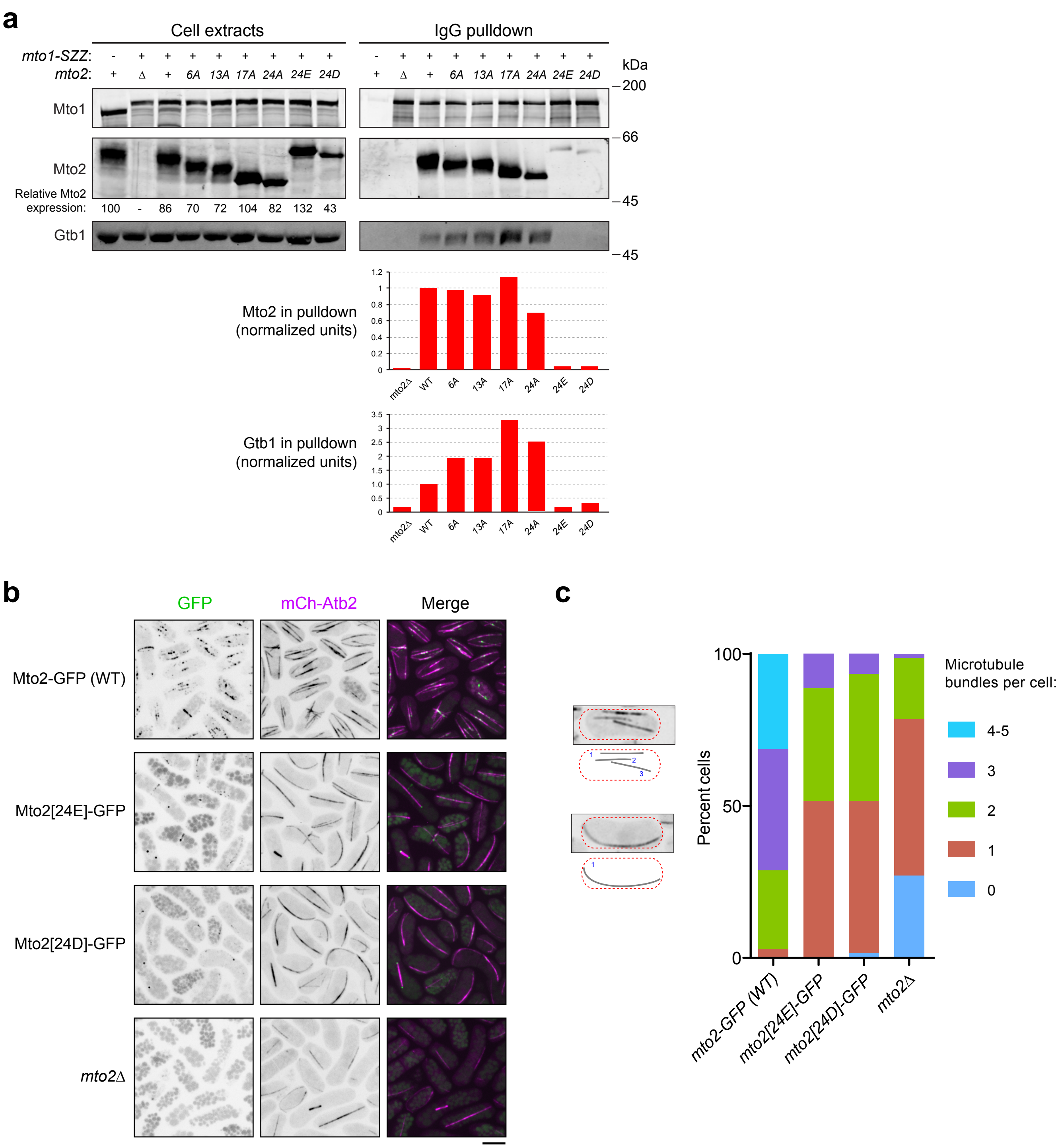

Supplementary Figure 8. Mutation of Mto2 phosphorylation sites to phosphomimetic glutamate or aspartate leads to loss of Mto1-Mto2 and Mto1/2-γ-TuC interactions, decreased Mto2-GFP puncta, and decreased numbers of cytoplasmic microtubules during interphase.
(a) Anti-Mto1, anti-Mto2 and Anti-Gtb1 (*S. pombe* γ-tubulin) Western blots of interphase cell extracts, and corresponding IgG pulldowns, from wild-type, *mto2-phosphomutant*, and *mto2-phosphomimetic* cells expressing Mto1-SZZ (Protein A tag). Mto2 expression levels are relative to the value for *mto1+ mto2+* cells (first lane, set to 100). Graphs show quantification of Mto2 (or phosphomutant/phosphomimetic Mto2) and Gtb1 co-purifying with Mto1-SZZ in the pulldowns above, normalized to the values obtained for wild-type (WT) cells (second column).
(b) Localization of GFP-tagged wild-type and phosphomimetic Mto2 as indicated, together with microtubule distribution (shown by mCherry-tubulin; mCh-Atb2). *mto2Δ* cells are also shown for comparison. Scale bar, 5 μm.
(c) Quantification of microtubule bundle number per cell, in the strains indicated, from images of the type shown in (b). For each strain, 70 cells were scored. Images and cartoons at left indicate method of quantification. P-values (Wilcoxon rank-sum test): *mto2-GFP(WT)* vs *mto2-phosphomimetic* and *mto2Δ*, p<1e-14; *mto2Δ* vs *mto2-phosphomimetic*, p<1e-5.

Supplementary Table 1: Mto2 phosphorylation sites and phosphosite mutations

aa	aa #	Sequence	Comment	Conserv'n	Net Phos predic'n	[S/T]P in homol. region	*mto2+* MS2 phos. prob.	*mto2 [17A]* MS2 phos. prob.	mut'd in *mto2[6A]*	mut'd in *mto2[13A]*	mut'd in *mto2[17A]*	mut'd in *mto2[NT1]*	mut'd in *mto2[24A]*	mut'd in *mto2[FCC]*	mut'd in *mto2[CT1]*	mut'd in *mto2[CT2]*
S	2	MSEHNY		Sz only	0.00			1.00				yes				
Y	6	SEHNYQSDR		-	0.91											
S	8	HNYQSDREV		-	0.79			0.50				yes				
Y	20	PFLNYEASA		Sz only	0.31			0.09								
S	23	NYEASANQL	plo	++	0.00			0.22				yes				
S	28	ANQLSSNSR		-	0.05			0.22				yes				
S	29	NQLSSNSRE	pbd	+	1.00			0.22				yes				
S	31	LSSNSREST		+++	0.99			0.22				yes				
S	34	NSRESTPRG		Sz only	1.00											
T	35	SRESTPRGS	TP PBD	-	1.00	++		N/A			yes	yes	yes	yes	yes	yes
S	39	TPRGSPWRA	CDK	+	0.99	++		N/A			yes	yes	yes	yes	yes	yes
S	47	AGMRSASLM		+	0.04								yes			
S	49	MRSASLMTE		+	0.89								yes			
T	52	ASLMTEPLE		+	0.04								yes			
S	58	PLEDSMYSD	PLO	+	0.15								yes			
Y	60	EDSMYSDNN		-/+	0.98											
S	61	DSMYSDNNY		-/+	0.07								yes			
Y	65	SDNNYLDNG		-/+	0.99											
S	71	DNGVSFTKD		+	0.06								yes			
T	73	GVSFTKDEN		-/+	0.29								yes			
Y	80	ENPLYSPSW		-	0.80											
S	81	NPLYSPSWP	SP	-/+	0.93	++	0.65	N/A		yes	yes	yes	yes	yes	yes	yes
S	83	LYSPSWPSL		-	0.02		0.70									
S	86	PSWPSLADA		+++	0.39		0.06									
S	94	ANVNSMKSN		-/+	0.46											
S	97	NSMKSNNAI		+	0.02			1.00								
S	111	AKFVSEKSL		-	0.97			0.06								
S	114	VSEKSLEKV	PLO	++	0.90			1.00						yes		
S	119	LEKVSTADN		+++	0.96			0.55						yes		
T	120	EKVSTADNN	pbd	+++	0.11			0.46						yes		
S	145	ELQLSERPS		+	0.87			0.33						yes		
S	149	SERPSSYLG		+	0.94		0.43	0.33						yes		
S	150	ERPSSYLGY	pbd	+++	0.98		0.54	0.33						yes		
Y	151	RPSSYLGYH		-	0.09		0.08	0.22								
Y	154	SYLGYHNNL		-/+	0.77											
S	159	HNNLSPYRS	CDK	+++	0.98	++	0.97	N/A	yes	yes	yes	yes	yes	yes	yes	yes
Y	161	NLSPYRSPN		Sz only	0.24		0.22									
S	163	SPYRSPNSY	SP	+	0.73	++		0.17								
S	166	RSPNSYPSL		-	0.60		0.13	1.00								
Y	167	SPNSYPSLL		-	0.52		0.18	0.11								
S	169	NSYPSLLPS		+	0.31		0.35	0.11								
S	173	SLLPSTHSP		-/+	0.33		0.37	0.11								
T	174	LLPSTHSPH	pbd	-/+	0.03		0.56	0.11								
S	176	PSTHSPHSP	SP	+	0.93	+	0.58	N/A		yes	yes	yes	yes	yes	yes	yes
S	179	HSPHSPAPL	SP	+	0.99	+	0.97	N/A		yes	yes	yes	yes	yes	yes	yes
S	184	PAPLSTMQT		-	0.80		0.14									
T	185	APLSTMQTA	pbd	-	0.01		0.21									
T	188	STMQTALMR		+++	0.12											
T	195	MRLRTYHPS		-/+	0.28											
Y	196	RLRTYHPSP		Sz only	0.04											
S	199	TYHPSPIIL	SP	Sz only	0.09	-										
T	215	NHAITLVNT		++	0.36											
T	219	TLVNTSPSS		+	0.06											
S	220	LVNTSPSSV	SP	+	0.96	-/+		N/A		yes	yes	yes	yes	yes	yes	yes
S	222	NTSPSSVVD		++	0.09											
S	223	TSPSSVVDA	pbd	++	1.00											
S	231	ALCRSLAEL		+++	0.11											
S	247	AIDASILSQ	PLO	-/+	0.01											
S	250	ASILSQQES		-/+	0.88											
S	254	SQQESSNSL		-/+	0.16											
S	255	QQESSNSLD	plo pbd	-/+	0.10											
S	257	ESSNSLDLV		-/+	0.03											
T	264	LVRHTPPLN	TP	+	0.17	++	1.00	N/A	yes	yes	yes	yes	yes	yes	yes	yes
Y	269	PPLNYTSSV		-	0.17		0.89	0.44							yes	
T	270	PLNYTSSVD	PLO	-/+	0.06		0.32	0.53							yes	
S	271	LNYTSSVDS		+	0.96		0.52	N/A		yes	yes	yes	yes	yes	yes	yes
S	272	NYTSSVDSS	pbd	-/+	0.05		0.84	0.34							yes	
S	275	SSVDSSPQR		-/+	0.64		0.49								yes	
S	276	SVDSSPQRM	CDK PBD	++	0.99	++	0.94	N/A	yes	yes	yes	yes	yes	yes	yes	yes
S	282	QRMASDSYG		++	0.98		0.44	N/A		yes	yes	yes	yes	yes	yes	yes
S	284	MASDSYGRP		-	0.99		0.56									
Y	285	ASDSYGRPS		Sz only	0.90		0.32									
S	289	YGRPSLHLN	ARK	+	0.98		0.44									
S	298	DPFPSVDLQ		+++	0.01			0.13								
S	303	VDLQSNELS		+	0.22			0.13								
S	307	SNELSHHNV	plo	+++	0.16			0.95								
T	313	HNVRTTLFS		-/+	0.02											
T	314	NVRTTLFSD	ARK	+	0.89			N/A		yes	yes	yes	yes	yes	yes	yes
S	317	TTLFSDDSR		-	0.34											
S	320	FSDDSRFHS	plo	-/+	0.27											
S	324	SRFHSKIHT		+	0.99											
T	328	SKIHTHSTP		-	0.03		0.27	0.62								
S	330	IHTHSTPPS		-/+	0.38		0.75	0.87								
T	331	HTHSTPPSQ	TP PBD	-/+	0.58	++	0.78	N/A	yes	yes	yes	yes	yes	yes	yes	yes
S	334	STPPSQMYS		+	0.10		0.84									
Y	337	PSQMYSAAS		-	0.08		0.06									
S	338	SQMYSAASH		-/+	0.03											
S	341	YSAASHFRY		-/+	0.98			N/A		yes	yes	yes	yes	yes	yes	yes
Y	345	SHFRYRSDP		-/+	0.26											
S	347	FRYRSDPST		-/+	0.85			0.42								
S	350	RSDPSTRHV	plo	-/+	0.79			0.29								
T	351	SDPSTRHVS	pbd	-	0.11			0.29								
S	355	TRHVSNSTN		-	0.92		0.05	0.56								
S	357	HVSNSTNKS		Sz only	0.80			0.75								
T	358	VSNSTNKSS	plo pbd	-	0.17			0.61								
S	361	STNKSSLHP	plo	-/+	0.05		0.75	0.47								
S	362	TNKSSLHPS	ARK pbd	-/+	0.78		0.94	0.10								
S	366	SLHPSPTSL	SP	+	0.96	++	0.95	N/A	yes	yes	yes	yes	yes	yes	yes	yes
T	368	HPSPTSLRV		-	0.26		0.33									
S	369	PSPTSLRVA		+	0.48		0.33									
S	382	PQRASPASQ	SP	+	0.99	++		0.99	yes							yes
S	385	ASPASQSFP		-/+	0.97			1.00								yes
S	387	PASQSFPSL		-/+	0.01			1.00								yes
S	390	QSFPSLQDT		-/+	0.99			0.10								yes
T	394	SLQDTPSP	TP	+	0.77	++		N/A			yes	yes	yes	yes	yes	yes
S	396	QDTPSP	SP	+	0.33	++		N/A			yes	yes	yes	yes	yes	yes

Comment: **SP, TP:** [S]-[P] or [T]-[P] (i.e. when not in strong CDK site). **CDK:** strong CDK site = [pS/pT]-P-X-[K/R]. **ARK:** strong AURORA site = [K/R]-X-[pS/pT]-[I/L/M/V] (Supplementary Refs. 5,6). **PLO:** strong Polo site = [D/E/N]-X-[pS/pT]-[F/I/L/M/W]. **plo:** weak Polo site = [D/E/N]-X-[pS/pT]-[no P] (Supplementary Refs. 7-9). **PBD:** strong Polo Box Domain binding site = S-[pS/pT]-P. **pdb:** weak Polo Box Domain binding site = S-[pS/pT] (Supplementary Ref. 10). ***Conservation:*** Qualitative assessment based on alignment shown in Supplementary Fig. 2, considering both extent of conservation among homologs and quaiity of alignment in the neighborhood. "Sz only" = conserved in *Schizosaccharomyces* species. ***NetPhos prediction:*** Probability score from NetPhos algorithm (Supplementary Ref. 11). ***[S/T]P in homologous region:*** Indicates if similar site is present nearby in homologs; see Supplementary Fig. 2 for details. ***MS2 Phosphorylation probabiilities:*** Summary of MS/MS analysis of Mto2 and Mto2[17A]; see Supplementary Data 1 for details. Red shading = high probabilities. Blue shading = low probabilities. N/A = not applicable (because mutated in Mto2[17A]). For residues grouped in double-lined boxes, at least one residue in box is phosphorylated, but MS2 data cannot make unambiguous assignment. In some cases, it is possible that some molecules of the relevant peptide are phosphorylated on one residue, but other molecules of the same peptide are phosphorylated on a different residue. For residues in thick-lined box (Ser355, Ser357, Thr358), peptide is doubly-phosphorylated, and thus at least two of the residues within the box are phosphorylated. Red and blue colors indicate high and low probabilities, respectively. ***Mut'd in:*** indicates which residues are mutated in the mutants listed.

Supplementary Table 2: Yeast strains used in this work

Strain	Genotype	Source
**KS516**	*h- ade6-M210 leu1-32 ura4-D18*	Lab stock
**KS1235**	*h+ kanMX:nmt81:GFP-atb2 ade6-M210 leu1-32 ura4-D18*	Lab stock
**KS1459**	*h+ mto2-GFP:kanMX mto1Δ::kanMX ade6-M216 leu1-32 ura4-D18*	Lab stock
**KS1740**	*h+ nda3-KM311 ade6-M216 leu1-32 ura4-D18*	Lab stock
**KS1890**	*h- mto2-GFP:kanMX ade6-M216 leu1-32 ura4-D18*	Lab stock
**KS3575**	*h+ mto1-SZZ:kanMX nda3-KM311 ade6-M216 leu1-32 ura4-D18*	Lab stock
**KS3953**	*h- mto2-HTB:kanMX ade6-M210 leu1-32 ura4-D18*	This study
**KS4323**	*h- mto1-SZZ:kanMX mto2Δ::kanMX ade6-M216 leu1-32 ura4-D18*	Lab stock
**KS5285**	*h- mto2-GFP:kanMX nda3-KM311 ade6-M216 leu1-32 ura4-D18*	Lab stock
**KS6404**	*h- mto2[13A]:hphMX mto1-SZZ:kanMX nda3-KM311 ade6 leu1-32 ura4-D18*	This study
**KS6407**	*h- mto2[13A]:hphMX kanMX:nmt81:GFP-atb2 ade6-M216 leu1-32 ura4-D18*	This study
**KS6412**	*h+ mto2[6A]:hphMX mto1-SZZ:kanMX nda3-KM311 ade6-M216 leu1-32 ura4-D18*	This study
**KS6415**	*h- mto2[17A]:hphMX nda3-KM311 ade6-M210 leu1-32 ura4-D18*	This study
**KS6512**	*h+ mto2[17A]:hphMX kanMX:nmt81:GFP-atb2 ade6-M216 leu1-32 ura4-D18*	This study
**KS6737**	*h+ mto2[6A]:hphMX kanMX:nmt81:GFP-atb2 ade6-M210 leu1-32 ura4-D18*	This study
**KS6741**	*h+ mto1-SZZ:kanMX mto2[17A]:hphMX nda3-KM311 ade6-M210 leu1-32 ura4-D18*	This study
**KS6760**	*h+ mto1-SZZ:kanMX cdc25-22 ade6-M216 leu1-32 ura4-D18*	This study
**KS6775**	*h+ alp4-GFP:hphMX natMX:Z:ADH15:mCherry-atb2 ade6-M210 leu1-32 ura4-D18*	Lab stock
**KS7055**	*h+ mto2-GFP:kanMX natMX:Z:ADH15:mCherry-atb2 ade6-M216 leu1-32 ura4-D18*	Lab stock
**KS7083**	*h- mto2[17A]-GFP:natMX nda3-KM311 ade6-M210 leu1-32 ura4-D18*	This study
**KS7093**	*h+ mto2[17A]-GFP:natMX car2Δ:kanMX arg1-230 lys3-37 nda3-KM311*	This study
**KS7099**	*h+ mto2[17A]-GFP:natMX ade6 leu1-32 ura4-D18*	This study
**KS7158**	*h+ mto2[17A]-GFP:natMX mto1Δ::kanMX ade6 leu1-32 ura4-D18*	This study
**KS7221**	*h+ mto2[FCC]:hphMX nda3-KM311 ade6-M210 leu1-32 ura4-D18*	This study
**KS7223**	*h+ mto2[NT1]:hphMX nda3-KM311 ade6-M210 leu1-32 ura4-D18*	This study
**KS7225**	*h+ mto2[CT1]:hphMX nda3-KM311 ade6-M210 leu1-32 ura4-D18*	This study
**KS7227**	*h+ mto2[24A]:hphMX nda3-KM311 ade6-M210 leu1-32 ura4-D18*	This study
**KS7229**	*h+ mto2[CT2]:hphMX nda3-KM311 ade6-M210 leu1-32 ura4-D18*	This study
**KS7413**	*h+ mto2[24A]-GFP:natMX:hphMX mto1Δ::kanMX ade6-M210 leu1-32 ura4-D18*	This study
**KS7417**	*h+ mto2[24A]-GFP:natMX:hphMX ade6-M210 leu1-32 ura4-D18*	This study
**KS7418**	*h- mto2[24A]-GFP:natMX:hphMX nda3-KM311 ade6-M210 leu1-32 ura4-D18*	This study
**KS7504**	*h- mto2[24A]-GFP:natMX:hphMX natMX:Z:ADH15:mCherry-atb2 ade6 leu1-32 ura4-D18*	This study
**KS7505**	*h+ mto2[17A]-GFP:natMX natMX:Z:ADH15:mCherry-atb2 ade6 leu1-32 ura4-D18*	This study
**KS7510**	*h+ mto2-GFP:kanMX mto1[NE]:kanMX cut12-tdT:kanMX ade6 leu1-32 ura4-D18*	This study
**KS7518**	*h+ natMX:nmt81:mto1[NE]:kanMX natMX:nmt81:mto2-GFP:kanMX ade6-M216 leu1-32 ura4-D18*	This study
**KS7525**	*h+ mto2[CT1]-GFP:natMX:hphMX ade6-M210 leu1-32 ura4-D18*	This study
**KS7534**	*h- mto2[FCC]-GFP:natMX:hphMX ade6-M210 leu1-32 ura4-D18*	This study
**KS7536**	*h- mto2[CT2]-GFP:natMX:hphMX ade6-M210 leu1-32 ura4-D18*	This study
**KS7541**	*h+ mto2[NT1]-GFP:natMX:hphMX ade6-M210 leu1-32 ura4-D18*	This study
**KS7542**	*h- mto2[24A]-GFP:natMX:hphMX mto1[NE]:kanMX cut12-tdT:kanMX ade6-M210 leu1-32 ura4-D18*	This study
**KS7545**	*h+ mto2[17A]-GFP:natMX mto1[NE]:kanMX cut12-tdT:kanMX ade6 leu1-32 ura4-D18*	This study
**KS7591**	*h- natMX:nmt81:mto1[NE]:kanMX natMX:nmt81:mto2-GFP:kanMX natMX:Z:ADH15:mCherry-atb2 ade6 leu1-32 ura4-D18*	This study
**KS7649**	*h+ mto2[24A]:hphMX kanMX:nmt81:GFP-atb2 ade6 leu1-32 ura4-D18*	This study
**KS7653**	*h+ mto1-SZZ:kanMX mto2[24A] nda3-KM311 ade6 leu1-32 ura4-D18*	This study
**KS7673**	*h+ natMX:nmt81:mto1[NE]:kanMX natMX:nmt81:mto2-GFP:kanMX alp4-tdT:natMX ade6-M216 leu1-32 ura4-D18*	This study
**KS7674**	*h+ mto2[24A]:hphMX alp4-GFP:hphMX natMX:Z:ADH15:mCherry-atb2 ade6-M210 leu1-32 ura4-D18*	This study
**KS7676**	*h+ mto2[17A]:hphMX alp4-GFP:hphMX natMX:Z:ADH15:mCherry-atb2 ade6-M210 leu1-32 ura4-D18*	This study
**KS7681**	*h- mto2[13A]-GFP:natMX:hphMX mto1Δ::kanMX ade6 leu1-32 ura4-D18*	This study
**KS7683**	*h+ mto2[13A]-GFP:natMX:hphMX ade6 leu1-32 ura4-D18*	This study
**KS7725**	*h+ mto2[6A]-GFP:hphMX ade6 leu1-32 ura4-D18*	This study
**KS7726**	*h+ mto2[6A]-GFP:hphMX mto1Δ::kanMX ade6 leu1-32 ura4-D18*	This study
**KS7736**	*h- mto1[bonsai]:kanMX natMX:nmt81:mto2-GFP:kanMX natMX:Z:ADH15:mCherry-atb2 ade6 leu1-32 ura4-D18*	This study
**KS7894**	*h+ mto1-SZZ:kanMX mto2[24E]:hphMX nda3-KM311 ade6-M216 leu1-32 ura4-D18*	This study
**KS7931**	*h- mto1-SZZ:kanMX mto2[24D]:hphMX nda3-KM311 ade6 leu1-32 ura4-D18*	This study
**KS7955**	*h+ plo1-mEGFP:kanMX pim1-F201S natMX:Z:ADH15:mCherry-Atb2 ade6 leu1-32 ura4-D18*	This study
**KS8002**	*h- mto2[24A]:hphMX plo1-mEGFP:kanMX pim1-F201S natMX:Z:ADH15:mCherry-atb2 ade6 leu1-32 ura4-D18*	This study
**KS8005**	*h- mto2[17A]:hphMX plo1-mEGFP:kanMX pim1-F201S natMX:Z:ADH15:mCherry-atb2 ade6-M210 leu1-32 ura4-D18*	This study
**KS8083**	*h+ mto2Δ::kanMX natMX:Z:ADH15:mCherry-atb2 ade6 leu1-32 ura4-D18*	This study
**KS8085**	*h+ mto2[24D]-GFP:natMX:hphMX natMX:Z:ADH15:mCherry-atb2 ade6 leu1-32 ura4-D18*	This study
**KS8091**	*h+ mto2[24E]-GFP:natMX:hphMX natMX:Z:ADH15:mCherry-atb2 ade6 leu1-32 ura4-D18*	This study

Supplementary Methods

Considerations underlying SILAC quantification of nonphosphopeptides

Peptides that are phosphorylated on multiple residues (here referred to as multiphosphopeptides) can be difficult to detect and/or identify experimentally, even after protein purification [12, 13]. For example, for the Mto2[17A] peptide sequence 381-A**S**PA**S**Q**S**FPSLQDAPAPR-398, we identified three distinct, high-confidence, singly-phosphorylated peptides (monophosphopeptides), each of which was phosphorylated on a different residue--one on S382, one on S385, and one on S387 (Supplementary Fig. 4, Supplementary Data 1)--but for the same sequence, we did not identify any di- or triphosphopeptides. These and similar results (e.g. Supplementary Fig. 3, Supplementary Data 1) illustrate the difficulty in detecting and identifying multiphosphopeptides with confidence, even when there is a good likelihood that they are present (although in principle our findings could also be due to mutually-exclusive monophosphorylation).

This may be particularly problematic for a protein such as Mto2[17A] (and more so for wild-type Mto2), because of the very high number of potential phosphorylation sites, and because a large proportion of Mto2 is predicted to be disordered. After excluding peptides containing five or fewer residues (which in general are too small to be uniquely identified) and taking account of incomplete cleavages, we determined that approximately 80% of Mto2[17A] tryptic peptides contain three or more serine or threonine residues, 70% contain four or more, and 50% contain five or more; percentages are marginally higher when tyrosine residues are also considered. Moreover, because of the high density of phosphorylation sites within Mto2, this situation is unlikely to be significantly improved by digestion with alternative proteases.

This raised the possibility that many Mto2[17A] peptides may be present as multiphosphopeptides *in vivo* but at the same time not easily detectable by mass spectrometry. We therefore sought to employ a method for SILAC-based relative quantification of phosphorylation that could overcome this obstacle. In the following example, we describe how two different approaches to quantifying the same experimental data can potentially lead to different conclusions, and we provide justification for why the second approach should be favored for analysis of Mto2[17A].

First, we consider that a given peptide of interest with multiple potential phosphorylation sites can exist in one of three states: nonphosphopeptide (denoted as “n”); monophosphopeptide (“p”); or multiphosphopeptide (“pp”). We further

consider that only the nonphosphopeptide and the monophosphopeptide can be detected; the multiphosphopeptide cannot be detected. We denote the true *in vivo* mole fractions of nonphosphopeptide, monophosphopeptide, and multiphosphopeptide in a "light" culture as "L_n", "L_p", and "L_{pp}", respectively. Similarly, mole fractions of nonphosphopeptide, monophosphopeptide, and multiphosphopeptide in a "heavy" culture are denoted as "H_n", "H_p", and "H_{pp}". By definition, $L_n + L_p + L_{pp} = 1.00$, and $H_n + H_p + H_{pp} = 1.00$. In the table below, we present hypothetical example values for these mole fractions for a hypothetical peptide that experiences a substantial increase in phosphorylation in the "heavy" culture relative to the "light" culture (e.g. in mitosis relative to interphase), accompanied by a shift towards multiphosphopeptides in the "heavy" culture:

**State of peptide**	**Mole fraction in "light" culture (e.g. interphase)**	**Mole fraction in "heavy" culture (e.g. mitosis)**
Nonphosphopeptide	$L_n = 0.90$	$H_n = 0.20$
Monophosphopeptide	$L_p = 0.10$	$H_p = 0.05$
Multiphosphopeptide	$L_{pp} = 0.00$	$H_{pp} = 0.75$
(Total peptide)	($L_n + L_p + L_{pp} = 1.00$)	($H_n + H_p + H_{pp} = 1.00$)

A first approach to quantification would involve simply determining the monophosphopeptide ratio in the "heavy" sample relative to the "light" sample:

$$\text{monophosphopeptide ratio} = \frac{H_p}{L_p}$$

While this is straightforward to determine, for the data in the example one obtains:

$$\text{monophosphopeptide ratio} = \frac{H_p}{L_p} = \frac{0.05}{0.10} = 0.5$$

By itself, this ratio would suggest that phosphorylation of the peptide is decreased in the "heavy" sample; however, this would be the opposite of the true trend of phosphorylation, because this approach does not take into account the (nondetectable) multiphosphopeptide. It is clear that this "false-negative" result is a direct consequence of the high mole fraction of multiphosphopeptide in the "heavy" sample; if this mole fraction were very small, then the "monophosphopeptide ratio" approach would in fact be suitable. However, *a priori* one can be sure of a low

multiphosphopeptide mole fraction only if the peptide of interest contains a single phosphorylatable residue, which is very rare within Mto2[17A]. More broadly, this approach also fails to give any indication of the stoichiometry of phosphorylation. For example, if L_p = 0.005 and H_p = 0.05, then the monophosphopeptide ratio H_p / L_p = 10, but the absolute level in phosphorylation may still be too low to be of biological significance. Finally, it is worth noting that if a given peptide were present as a multiphosphopeptide but not as a monophosphopeptide, then it would not even be possible to determine a monophosphopeptide ratio.

As an alternative to calculating monophosphopeptide ratio, one can follow a second approach, namely to quantify the nonphosphopeptide ratio in the “light” sample relative to the “heavy” sample [14]:

$$\text{nonphosphopeptide ratio} = \frac{L_n}{H_n}$$

For the data in the example (i.e., in which the combined mole fraction of monophosphopeptide and multiphosphopeptide is high), one obtains:

$$\text{nonphosphopeptide ratio} = \frac{L_n}{H_n} = \frac{0.90}{0.20} = 4.5$$

With this approach, one identifies a substantial difference in nonphosphopeptide abundance that is consistent with the true trend of phosphorylation. The main disadvantage of this method in screening for phosphorylation sites is that it presumes that a decrease in the mole fraction of nonphosphopeptide in the “heavy” sample is specifically due to phosphorylation, when in fact such a decrease could be due to any modification (or, alternatively, proteolytic processing *in vivo*, resulting in the loss of an N- or C-terminal region--although in our case, purified Mto2[17A]-GFP showed similar migration on SDS-PAGE for “heavy” and “light” samples). As a result, this approach is likely to result in the greatest number of “false-positive” results. However, if the approach is further validated by direct demonstration of phosphorylation of the relevant peptide or, as shown in this work, demonstrated changes in protein phosphorylation (i.e. by Phos-tag Western blot) after mutation of the relevant residues, then it can be useful as a “first-pass” screening tool to identify protein regions subject to post-translational modification. In addition, a key advantage of quantifying nonphosphopeptides is that the ratios obtained should be at least approximately related to the stoichiometry of modification (again, with the presumption that any measured difference is due to only one type of modification).

Thus, when a modification of interest is expected to be present at relatively high stoichiometry (as is suggested, in our case, from Western blot data comparing Mto2[17A] migration in interphase vs. mitosis), this second approach will likely result in the smallest number of “false-negative” results. Finally, it is worth noting more generally that when the only two forms of a given peptide are nonsphosphopeptide and monophosphopeptide (i.e. multiphosphopeptide mole fraction is very low or zero), then SILAC quantification of nonphosphopeptide and monophosphopeptide together can be used to determine phosphorylation site occupancy much more accurately [15].

Supplementary References

1. Linding R, Russell RB, Neduva V, Gibson TJ. GlobPlot: Exploring protein sequences for globularity and disorder. *Nucleic Acids Res* **31**, 3701-3708 (2003).

2. Altschul SF*, et al.* Gapped BLAST and PSI-BLAST: a new generation of protein database search programs. *Nucleic Acids Res* **25**, 3389-3402 (1997).

3. Sievers F*, et al.* Fast, scalable generation of high-quality protein multiple sequence alignments using Clustal Omega. *Mol Syst Biol* **7**, 539 (2011).

4. Holt LJ, Tuch BB, Villen J, Johnson AD, Gygi SP, Morgan DO. Global analysis of Cdk1 substrate phosphorylation sites provides insights into evolution. *Science* **325**, 1682-1686 (2009).

5. Alexander J*, et al.* Spatial exclusivity combined with positive and negative selection of phosphorylation motifs is the basis for context-dependent mitotic signaling. *Sci Signal* **4**, ra42 (2011).

6. Cheeseman IM*, et al.* Phospho-regulation of kinetochore-microtubule attachments by the Aurora kinase Ipl1p. *Cell* **111**, 163-172 (2002).

7. Burkard ME*, et al.* Plk1 self-organization and priming phosphorylation of HsCYK-4 at the spindle midzone regulate the onset of division in human cells. *PLoS Biol* **7**, e1000111 (2009).

8. Santamaria A*, et al.* The Plk1-dependent phosphoproteome of the early mitotic spindle. *Mol Cell Proteomics* **10**, M110 004457 (2011).

9. Oppermann FS, Grundner-Culemann K, Kumar C, Gruss OJ, Jallepalli PV, Daub H. Combination of chemical genetics and phosphoproteomics for kinase signaling analysis enables confident identification of cellular downstream targets. *Mol Cell Proteomics* **11**, O111 012351 (2012).

10. Elia AE, Cantley LC, Yaffe MB. Proteomic screen finds pSer/pThr-binding domain localizing Plk1 to mitotic substrates. *Science* **299**, 1228-1231 (2003).

11. Blom N, Gammeltoft S, Brunak S. Sequence and structure-based prediction of eukaryotic protein phosphorylation sites. *J Mol Biol* **294**, 1351-1362 (1999).

12. Ishihama Y, Wei FY, Aoshima K, Sato T, Kuromitsu J, Oda Y. Enhancement of the efficiency of phosphoproteomic identification by removing phosphates after phosphopeptide enrichment. *J Proteome Res* **6**, 1139-1144 (2007).

13. Thingholm TE, Jensen ON, Larsen MR. Analytical strategies for phosphoproteomics. *Proteomics* **9**, 1451-1468 (2009).

14. Singh S, Springer M, Steen J, Kirschner MW, Steen H. FLEXIQuant: a novel tool for the absolute quantification of proteins, and the simultaneous identification and quantification of potentially modified peptides. *J Proteome Res* **8**, 2201-2210 (2009).

15. Olsen JV*, et al.* Quantitative phosphoproteomics reveals widespread full phosphorylation site occupancy during mitosis. *Sci Signal* **3**, ra3 (2010).
